# A Wavelength Rule for the Analysis of Clusteroluminescence

**DOI:** 10.3390/polym17141908

**Published:** 2025-07-10

**Authors:** Frank B. Peters, Andreas O. Rapp

**Affiliations:** Institut für Berufswissenschaften im Bauwesen, Leibniz-Universität Hannover, Herrenhäuser Straße 8, 30419 Hannover, Germany

**Keywords:** clusteroluminescence, through-space interaction, excitation-emission correlation, red-shift, polymer luminescence, excitation-dependent emission, wood fluorescence

## Abstract

A key discovery of this study is the strong correlation (r = 0.96) between excitation and emission maxima across chemically distinct clusteroluminogens. All 157 evaluated peaks fall along a single regression line (Ex = 0.844 Em − 12 nm), a pattern that was not valid for conventional fluorophores. This suggests a general principle of clusteroluminescence. We show that in lignocellulosic materials, peak positions reflect chemical interactions: isolated lignin and cellulose showed short excitation and emission wavelengths, while native wood exhibited longer wavelengths. Fungal or photoinduced degradation led to a further red-shift. These effects are attributed to increased molecular heterogeneity, reducing the effective energy gap within the lignocellulosic complex. We conclude that the spectral position reflects the degree of molecular interaction rather than the chemical structure of individual molecules. It may serve as a novel analytical parameter for assessing purity and degradation in a wide range of polymers.

## 1. Introduction

In recent years, clusteroluminogens have gained increasing attention. This new class of luminogens shows properties that help overcome typical problems of conventional fluorophores. Many clusteroluminogens are organic, biobased and biodegradable, abundant, cheap, non-toxic, water-soluble, and light-stable. They have huge potential for a wide range of applications, including as nanosensors for ecosystem monitoring, markers in medical systems, materials for optoelectronics, encryption, and forensics [1,2,3,4,5].

The physical principles underlying clusteroluminescence remain widely discussed [6,7]. While in conventional fluorescence, extended π-electron conjugation is considered a primary mechanism [8], the emission mechanism of clusteroluminescence is substantially different. It is widely accepted that clusteroluminescence originates from the spatial clustering of non-conjugated functional groups (e.g., hydroxyl, carbonyl, amino, or ester groups). These clusters form non-covalent through-space interactions that allow intra- and intermolecular electron delocalization [9,10,11,12]. As a consequence, clusteroluminescence arises from interactions and cannot be ascribed to single molecules. The current state of the scientific discussion was recently reviewed by Zhao et al. [7].

Interestingly, this phenomenon also occurs in naturally abundant polymeric materials such as lignocellulose in wood. For decades, wood was regarded as non-luminescent, except for a few strongly fluorescent species [13,14], where the fluorescence is caused by specific soluble fluorophores with conventional molecular fluorescence. These rare exceptions must be carefully distinguished from the weak native blue luminescence of lignin [15,16], cellulose [17,18] and other polysaccharides [19,20], which is consistently present in all wood species [21,22] and can be shifted by various modifications of the wood chemistry [22,23,24].

The native luminescence existing in all wood species can be attributed to clusteroluminescence. This conclusion is supported by the fact that wood shows the key characteristics that distinguish clusteroluminogens from fluorophores, as summarised by Zhang et al. [25]:

- Polysaccharides and lignin do not contain extended conjugated π-electron systems that could explain luminescence in the visible range [26,27].
- Their luminescence is greatly enhanced in the solid state, but negligible in dilute solutions [26], also known as aggregation-induced emission.
- Their excitation maxima lie at longer wavelengths than the absorption maxima [28,29].
- Their emission maxima depend on the excitation wavelength, shifting to longer wavelengths with increased excitation wavelength [30,31,32]. This excitation-dependent emission is a violation of Kasha’s rule, a basic principle in fluorescence [33].

These characteristics of wood clusteroluminescence introduce methodological requirements, which have often been unaddressed in earlier studies. The majority of studies start from well-established rules in fluorescence analysis, like the similarity of absorption and excitation spectra, and the independence of emission wavelength from excitation. Accordingly, the absorption maximum is often chosen as the single excitation wavelength, which does not reveal the true emission maximum in systems with excitation-dependent emission. In conclusion, full excitation–emission matrices (EEMs) are necessary to properly characterise clusteroluminescence.

This study takes wood and its major compounds as starting points for exploring clusteroluminescence. By providing corresponding EEM data and placing them in the context of other clusteroluminogens, we identify commonalities and differences in their photophysical behaviour. To the best of our knowledge, this is the first study to establish a generalizable linear relationship between excitation and emission maxima in chemically distinct clusteroluminogens. Additionally, we identify molecular heterogeneity as a common factor of peak shifts, and discuss its applicability as a new analytical parameter to assess purity and degradation in polymers, beyond the context of lignocellulose.

## 2. Material and Methods

### 2.1. Samples

In an exploratory approach, a broad range of lignocellulosic materials were selected and grouped into three categories: untreated wood, degraded wood, and isolated compounds including related biopolymers:

(1) In the unmodified class, 14 light-coloured wood species were chosen to reduce the effect of reabsorption: *Abies alba*, *Acer* ssp., *Alnus glutinosa*, *Betula pendula*, *Carpinus betulus*, *Fagus sylvatica*, *Fraxinus excelsior*, *Ochroma* ssp., *Picea abies*, *Prunus avium* sapwood, *Terminalia superba*, *Tilia* ssp., and *Triplochiton scleroxylon*.
(2) The degraded wood samples included *Fagus sylvatica* wood degraded by *Trametes versicolor*, *Picea abies* wood degraded by *Serpula lacrymans*, and photodegraded *Acer* ssp. wood.
(3) The isolated and related lignocellulosic materials comprised bacterial cellulose, cellulose for column chromatography, cotton nettle, chitin from shrimp shells, fluffy mycelium of *Serpula lacrymans*, D (+) Xylose, filter paper, microcrystalline cellulose with varying moisture content, and organosolv lignin from *Eucalyptus* ssp. A detailed list of sample origin and treatments is given in the Supplementary Materials. All samples were stored in the dark at room temperature until measured, unless otherwise stated.

### 2.2. Fluorescence Spectroscopy

All solid samples were analysed in a front-face configuration using a F-7100 spectrofluorometer (Hitachi, Tokyo, Japan) equipped with a solid sample holder and an additional light path-modifying device described elsewhere [34]. The spectra were corrected according to the specifications of the manufacturer, and a 20 nm moving-average smoothing was applied to the calibration curve. Powder samples were filled in a PMMA cuvette and measured in the same way as the other solid samples. Fluid and dispersed samples were analysed using a quartz cuvette in 90° configuration. Data collection was performed with the Hitachi FL Solutions software (Version 4.2). The spectral correction and the removal of scattering signals was carried out in MATLAB (Version R2023A) using the EEM filtering tool in the PLS Toolbox (Version 9.0, Eigenvector Research Inc., Manson, WA, USA).

### 2.3. Literature Analysis

A total of 120 excitation maxima (Ex) and emission maxima (Em) of various clusteroluminogens were compiled from the literature. To exclude conventional fluorescence, only peaks that met key criteria of clusteroluminescence were included, with criteria listed below:

(1) An absence of extended conjugated structures able to explain conventional fluorescence.
(2) An excitation maximum substantially different from the absorption maximum.
(3) Excitation-dependent emission.

These clusteroluminogens encompassed a broad range of chemical classes, including the following:

- Polysaccharides [35,36,37,38,39,40,41,42,43,44,45],
- Lignin [35,45],
- Proteins [37,46,47,48,49,50,51,52,53],
- Small organic molecules [54,55],
- Synthetic polymers [56,57,58,59,60,61], and
- Polymer dots or carbon dots [62,63,64,65,66,67].

This variety of chemical substances contained some well-defined systems based on different clustering moieties like hydroxyls and ethers (e.g., cellulose), amines and carbonyls (e.g., Poly-L-lysine), hydroxyls (e.g., polyvinyl alcohol), esters (aliphatic polyesters), oxime groups [55], nitrils [61], and a number of less-well-defined systems and natural products (e.g., white hair or unbleached pulp) with combinations of the mentioned clustering moieties. It spanned systems with low and high molecular weight. At this exploratory stage, the selection was not intended to proportionally represent clusteroluminogens of all classes, but to include samples from a broad range of fields. A detailed list of substances, peak positions, and measurement conditions is provided in the Supplementary Materials. These clusteroluminogens were compared with a reference dataset of conventional fluorophores [68].

## 3. Results

**Figure 1:**
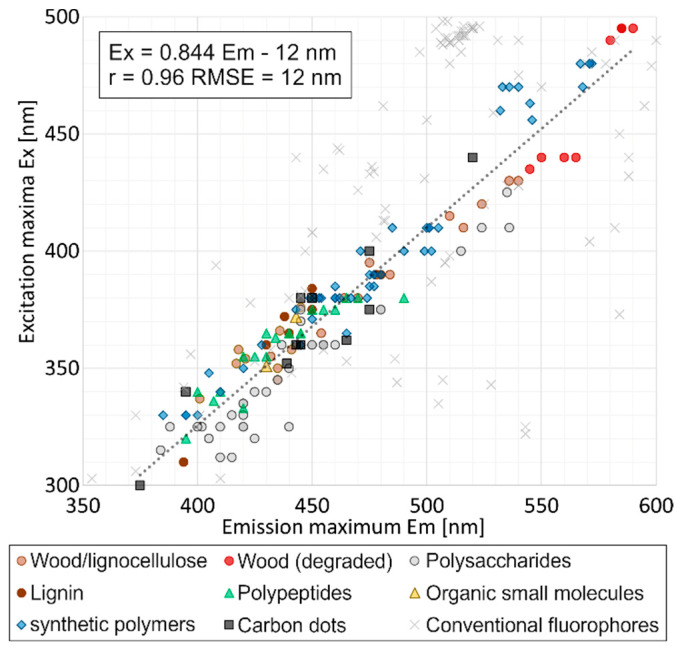
Clusteroluminescence peak positions of all excitation and emission maxima included in this study. For comparison, conventional fluorophores [68] are shown in pale grey. A detailed list of the substances and references is provided in the Supplementary Materials.

Across all spectra included in this study, we found a strong correlation between the wavelengths of excitation and emission maxima. This included own measurements and literature spectra of lignocellulosics, proteins, synthetic polymers, organic small molecules, and polymer or carbon dots (Figure 1). The correlation coefficient was r = 0.96. A linear regression analysis yielded Equation (1):

Ex = 0.844 Em − 12 nm(1)

where Ex is the excitation maximum and Em is the emission maximum of the respective peak. The root mean squared error (RMSE) of these clusteroluminogens was 12 nm. In contrast, the projection of a set of conventional fluorophores [68] onto the regression line yielded an RMSE of 89 nm, indicating a rather random distribution. It can be safely concluded that Equation (1) reflects a general principle in clusteroluminescence, while it is not valid for conventional fluorophores.

**Figure 2:**
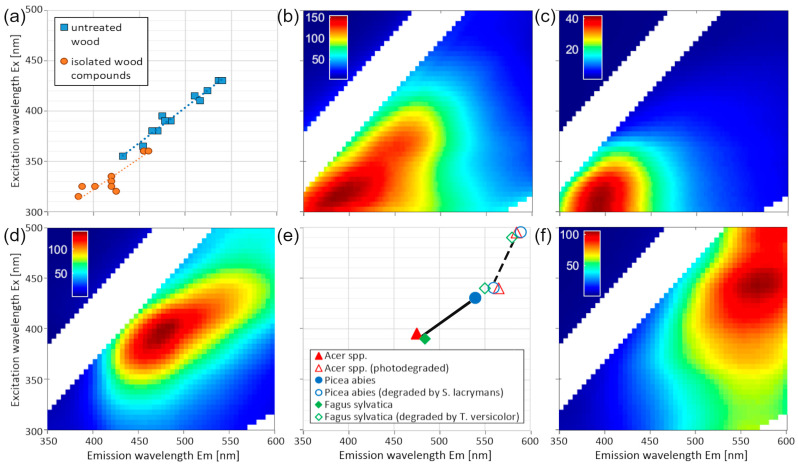
(**a**) Peak positions of all examined wood species and isolated lignocellulosic compounds. (**b**) Excitation–emission matrix (EEM) of solid bacterial cellulose. (**c**) EEM of 0.1% organosolv lignin of *Eucalyptus* ssp. in 60% ethanol. (**d**) EEM of untreated *Acer* ssp. Wood. (**e**) Peak positions of wood species before (solid line) and after (broken line) various degradations. (**f**) EEM of photodegraded *Acer* ssp. wood.

The lignocellulosics measured in this study also grouped along this regression line, with an RMSE of 14 nm. When measured in pure form, these chemical compounds of wood peaked at the lower end of the regression line, with Ex 310–360 nm and Em 388–460 nm (Figure 2a). Representative EEMs of isolated cellulose and lignin are shown in Figure 2b and Figure 2c, respectively.

In contrast, the wood samples showed luminescence at higher wavelengths (Ex = 355–430 and Em = 432–540), with the absence of peaks of the isolated wood compounds (Figure 2a). An example EEM of *Acer* ssp. is displayed in Figure 2d, with a peak at Ex 395/Em 475 nm.

In degraded wood samples (Figure 2e), the luminescence was further red-shifted, regardless of the type of degradation. An EEM of photodegraded *Acer* ssp. is exemplarily shown in Figure 2f, where the peak was red-shifted to Ex 440/Em 565 nm.

## 4. Discussion

### 4.1. Correlation Between the Wavelengths of Excitation and Emission Maxima

The most striking observation in this study was the strict correlation between the wavelengths of excitation and emission maxima. The fact that this holds across chemically very different organic polymers suggests a new principle, contrary to conventional fluorescence (Figure 1). To the best of our knowledge, this has not been reported before.

We therefore propose to add the following point to the common features of clusteroluminogens as listed by Zhang et al. [25]: “By empiric evidence, the wavelengths of the excitation (Ex) and emission (Em) maxima of clusteroluminogens follow a consistent relationship (r = 0.96), where the excitation maximum can be estimated by Equation (1).”

In order to better reflect the underlying molecular phenomena, the wavelengths of excitation and emission maxima were converted into electron volts (eV), and a new regression analysis was performed in energy space. For theoretical consistency and simplicity, the regression was constrained to pass through the origin. This yielded Equation (2):

E_Ex_ = 1.224 × E_Em_ (2)

where E_Ex_ and E_Em_ are the excitation and emission energies, respectively. This means that on average, the excitation energy of clusteroluminogens is 22.4% higher than their emission energy. The regression performance (r = 0.95; RMSE = 0.10675 eV) was comparable to the wavelength-based model described above.

For comparison, an unconstrained energy-based regression (i.e., one that allowed a non-zero intercept) produced an offset of −0.039 eV and an RMSE of 0.10668 eV. Given the small offset and the virtually identical RMSE, forcing the regression through the origin is well justified. As a consequence, the relationship between excitation and emission energy levels can be regarded not only as linear, but even as proportional.

The variation in this study is partially explained by the fact that spectral correction was only applied in part of the reviewed studies, and values were recorded on different instruments. Both instrumental spectrum correction and inter-instrumental variations (even after correction) are reported to highly contribute to statistical error of fluorescence spectra [69]. Therefore, an RMSE of 12 nm is within expectations. An analysis of the residuals (predicted vs. measured values) showed that residuals were approximately normally distributed (Shapiro–Wilk W = 0.988, *p* = 0.178), but variance slightly increased with emission wavelength, indicating heteroskedasticity (Breusch-Pagan *p* = 0.010). Thus, not all deviations from the above equations could completely be explained by instrumental error. Different classes of substances seemed to group left or right of the regression line (Figure 1).

The close correlation and the fact that it applies to a great variety of substances are a strong indication of an underlying energetic principle of through-space interactions in clusteroluminogens. Within this exploratory study, we provide a robust empirical observation that may inspire future research and detailed mechanistic explanations.

### 4.2. Analytical Interpretation of the Peak Position on the Regression Line

In a recent review, Zhao et al. [7] identified three key components of clusteroluminescence: (1) the presence of electron-rich moieties like hydroxyls, carbonyls, amines, amides etc., (2) their clustering, and (3) conformational rigidity of the clusters. Therefore, clusteroluminogens are generally sensitive to their surrounding environments, such as viscosity, temperature, pH, etc.

Zhang et al. [25] proposed that the “chromophore” in clusteroluminogens is built on inter- and intramolecular through-space conjugation. From isolated to linked and further to clustered forms, the energy gap decreases, resulting in a red-shifted clusteroluminescence. Based on that, cluster size is widely accepted to influence the emission wavelength of clusteroluminogens, where “size” refers to the molecular weight of polymers, diameter of nanoparticles, generations of dendrimers, etc. [25].

Our interpretation of the peak shifts measured in this study is that not only cluster size, but structural heterogeneity (in particular with regard to functional groups) within a material effectively reduce the energy gap and are therefore responsible for the observed peak shifts. This conclusion is based on the following observations.

Across the lignocellulosic samples measured in this study, we observed a continuous red-shift with increasing structural heterogeneity within the materials. Bacterial cellulose, the purest form of cellulose [70] without residues of lignin, exhibited the shortest Ex and Em maxima of 315 and 384 nm, respectively (Figure 2b). Cotton nettle, as an example for a less pure sample, showed two peaks at longer excitation and emission wavelengths (335/425 and 360/460 nm; see Supplementary Materials).

Furthermore, the typical peaks observed in isolated polysaccharides and lignin did not appear as distinct peaks or even as shoulders in the spectra of wood, and not at all in degraded wood. For example, cellulose is known to be relatively stable against photodegradation [71], but its peaks were not observed in photodegraded wood (Figure 2f), not even as remnants. We explain this by a greater structural heterogeneity in degraded wood, i.e., the introduction of new electron-rich entities (e.g., carbonyl groups). These new entities interact with the existing ones, forming new energy levels and thereby effectively decreasing the energy gap.

Remarkably, the observed shifts were similar across the assessed fungal and photodegradation processes, although different wood compounds were degraded. While photodegradation preliminary degrades lignin [71], *Serpula lacrymans* selectively metabolises the polysaccharides in wood [72]. *Trametes versicolor* attacks both lignin and polysaccharides [71]. The chance that all three processes produce the same fluorophores is low. A common feature of these processes is that they are all oxidative and foster the formation of carbonyl and carboxyl groups [21,71,73], but in different parts of the wood. Both carbonyl and carboxyl groups are known to participate in cluster formation [6]. This underlines the understanding of clusteroluminescence as a consequence of interactions between electron-rich entities rather than individual fluorophores.

To critically evaluate our interpretation of the structural heterogeneity as a factor driving peak shifts in clusteroluminogens, we evaluated factors associated with wavelength shifts reported in the literature. A brief synthesis is presented in Table 1.

Most of the reported red-shifts can be satisfactorily explained by the introduction of additional accessible energy levels decreasing the effective energy gap. In the upper part of Table 1, this involves introduction of new functional groups or heteroatoms (e.g., O, N, or S) into existing systems. This is referred to as increased structural heterogeneity above. The same effect (additional energy levels) can be achieved by rearrangement of existing structures as in the lower part of Table 1. The increase in packing density (like in planar folded structures) brings isolated moieties closer together and increases the number of accessible energy levels. Blue-shifts, on the contrary, mostly involve the decrease in accessible energy levels. This translates to removal of impurities in the upper part of Table 1, and reduction in cluster size or isolation of clusters (e.g., by dissolution of solids) in the lower part.

Taken together, the reviewed literature supports the hypothesis that structural heterogeneity is a key factor driving the observed red-shifts in clusteroluminescence. The introduction of new electron-rich groups increases the heterogeneity of clusteroluminescent entities. As a result, additional energy levels are formed, decreasing the energy gaps for through-space interaction. By contrast, isolated compounds (amino acids, polysaccharides, and lignin fragments) exhibit relatively short wavelength emissions, consistent with their lower structural heterogeneity.

For specific systems, the effects of heterogeneity require experimental validation. Investigations using model substances will be addressed in future studies.

### 4.3. Implications for Analytical Methods

Established spectral decomposition methods such as Parallel Factor Analysis (PARAFAC) have been highly successful in resolving dilute solutions of fluorophores in complex systems [93,94,95]. However, the properties of clusteroluminescence present a unique challenge and a major disruptive factor in fluorescence spectra, especially in concentrated or solid samples. While conventional factorization models assume independent spectral components with fixed excitation and emission spectra, they cannot be directly applied to the analysis of clusteroluminescence. Our results highlight the following key limitations that must be considered when analysing clusteroluminescence:

- Variability in peak positions: The same substance can contribute to both short- and long-wavelength luminescence along the regression line (Figure 1), depending on its molecular environment (e.g., cellulose in pure form, in wood, or in degraded wood). This means that there is no specific peak position for one chemical compound in mixtures of clusteroluminogens.
- Reduced number of independent variables: Since the excitation and emission maxima are not independent from each other (see Equation (1)), the informative value of excitation-emission matrices (EEMs) is effectively reduced by one dimension. This decreases the specificity of EEMs when analysing clusteroluminogens.
- Violation of Kasha’s rule: Unlike conventional fluorophores, clusteroluminogens emit from multiple emissive states, resulting in excitation-dependent emission [10]. The record of one excitation and one emission spectrum does therefore not reveal the true shape of a clusteroluminescence peak.
- No linear correlation between concentration and intensity: Since clusteroluminogens emit best in a concentrated and solid state, they cannot be dissolved or diluted without affecting their emission properties.

These challenges complicate the use of established trilinear algorithms in systems with extensive clusteroluminescence and underline the need for a novel analytical perspective. A fundamental research opportunity consists in the further development of shift-invariant factorization models like SIT [96] or complementary analytical approaches to better capture the unique spectral properties of clusteroluminescence.

**Figure 3:**
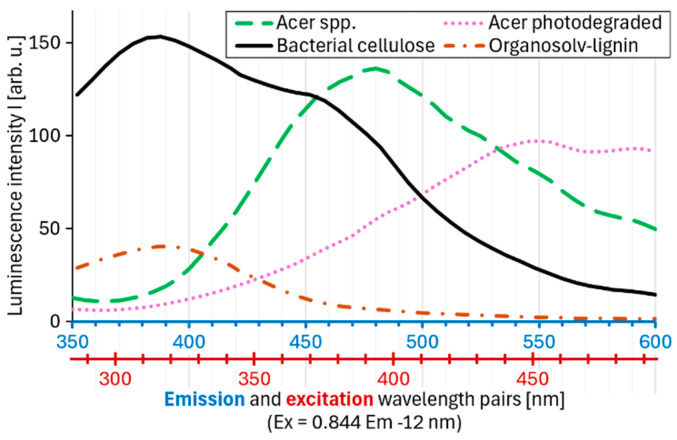
Proposed two-dimensional spectrum for clusteroluminogens with fixed pairs of excitation and emission wavelength, based on Equation (1). With this approach, all clusteroluminescence peaks of the four displayed samples were captured. For comparison, full EEMs can be found in Figure 2.

As a first conceptual attempt especially suited for clusteroluminescence, we propose that the peak position along the excitation-emission regression line (Figure 1) should be considered a new analytic parameter, which provides information about the purity, heterogeneity, and cluster size of luminescent species within a material. Similarly to synchronic fluorescence spectroscopy, excitation and emission wavelengths could be scanned simultaneously. Instead of a fixed offset, however, Equation (1) would be the underlying rule. Figure 3 shows such a spectrum.

In this way, most relevant information from full EEMs is retained, while greatly reducing the amount of data. This approach offers several advantages:

- Faster spectra acquisition: Instead of acquiring a full EEM, excitation and emission wavelengths can be scanned simultaneously.
- Little to no sample preparation needed: Since clusteroluminogens emit best in the solid or concentrated state, clusteroluminescence enables direct measurement in front-face mode. This allows rapid and non-destructive analysis for industrial in-line and on-line applications.

Potential applications are widespread, including industrial process control, life sciences, material quality assessment, and the monitoring of ageing and degradation processes in polymers. In addition to spectroscopic techniques, the reduced dimensionality of the concept may inspire new imaging strategies. For instance, multispectral and luminescence imaging systems, from microscopy to large-scale sensing, could be adapted to specifically capture the important excitation/emission combinations of clusteroluminogens and display the peak position in a simple colour scale, spatially resolved per each pixel of an image.

## 5. Conclusions

The found regression line as a specific feature of clusteroluminogens may assist researchers in differentiating between conventional fluorescence and clusteroluminescence. In polymer analysis, it offers a potential tool for assessing clustering behaviour, material purity, ageing and other structural modifications without the need for full EEMs. Future research should refine this approach, integrate it into industrial monitoring systems, and explore its applicability beyond lignocellulosics.

## Figures and Tables

**Table 1:** Factors associated with wavelength shifts in clusteroluminogens collected from the literature.

	Factor	Shift	Substance	Ref.
Chemical changes in the structural heterogeneity	Thermal ageing	Red	Proteins	[74]
	Thermal degradation (200 to 400 °C)	Red	Poly (acrylic acid)	[75]
	Ageing by lifetime and photoageing	Red	Skin (mouse)	[76]
	Glycation (Maillard reaction) and oxidation	Red	Proteins, advanced glycation end products	[77,78,79]
	Photodegradation	Red	Wood	[21]
	Introduction of amino groups into an oxygenic clustering system	Red	Lignin	[80,81]
	Introduction of N and S atoms into an oxygenic clustering system	Red	Aliphatic polyesters	[82,83]
	Hydrothermal treatment	Red	Lignin	[84,85]
	Increased variety of monomers used for copolymerisation	Red	Dehydrogenated polymer from 100% coniferyl alcohol compared to 50% coniferyl alc. with 50% sinapyl alc.	[35]
	H_2_O_2_ bleaching (=reduction of carbonyl groups)	Blue	Mechanical pulp	[17,86]
	Removal of impurities	Blue	Dithiosuccinimide	[87]
Changes in interactions between existing cluster entities	Change of secondary structure (helix to straight to planar folded)	Red	Aliphatic polyesters	[56]
	Increase in degree of polymerisation	Red	Poly (maleic anhydride-alt-vinyl pyrrolidone)	[58]
	Increase in degree of polymerisation	Red	Oligo-L-alanine vs. poly-L-alanine	[88]
	Reversible molecular rearrangement (by light)	Red	Dimethyl terephthalate	[89]
	Ball milling from nanofibers to particles	Red	Cellulose nanofibers and nanoparticles	[28]
	Increase in pH (maximum Em at pH10)	Red	Carboxy-nanocellulose	[90]
	Transfer to solvents with electron-rich atoms	Red	Poly (maleic anhydride-alt-vinyl acetate)	[91]
	Crystallisation from different solvent (polymorph)	Red/Blue	Furan-maleic anhydride and furan-maleimide	[92]
	Dissolution of solids	Blue	Rice, starch, cellulose	[38]
	Dissolution of solids	Blue	Gelatin	[53]
	Dissolution of solids	Blue	Maleimide and succinimide	[87]
	Dissolution of solids	Blue	Aliphatic polyesters	[56]
	Steam explosion	Blue	Various lignocellulosics	[35]
	Moisture swelling	Blue	*Pinus sylvestris* wood	[22]

## Data Availability

The original data presented in the study are openly available in FigShare (http://www.doi.org/10.6084/m9.figshare.29314412).

## Supplementary Materials

The following supporting information can be downloaded at https://www.mdpi.com/article/10.3390/polym17141908/s1, Table S1: Origin and treatment of examined samples. Table S2: Peak positions of the clusteroluminogens. Table S3: Peak positions of the conventional fluorophores, from [68]. Figures S1–S29: EEMs of examined samples.
