# Supplementary material for: A Wavelength Rule for the Analysis of Clusteroluminescence
Source: Polymers (Basel). 2025 Jul 10;17(14):1908. doi: 10.3390/polym17141908 (PMC12300059; doi:10.3390/polym17141908)
Supplement: Supplementary file 1 [file polymers-17-01908-s001.zip › polymers-3728415-supplementary.pdf]

A wavelength-rule for the Analysis of Clusteroluminescence

Supplementary material

Frank B. Peters and Andreas O. Rapp

Table S1: *Origin and treatment of examined samples*

Untreated wood samples			
**Nr.**	**Sample**	**Source**	**Treatment**
01	*Abies alba*	Local trader	None
02	*Acer ssp.*	Local trader	None
03	*Alnus glutinosa*	Local trader	None
04	*Betula pendula*	Local trader	None
05	*Carpinus betulus*	Local trader	None
06	*Fagus sylvatica*	Local trader	None
07	*Fraxinus excelsior*	Local trader	None
08	*Ochroma ssp.*	Local trader	None
09	*Picea abies*	Local trader	None
10	*Prunus avium sapwood*	Local trader	None
11	*Terminalia superba*	Local trader	None
12	*Tilia ssp.*	Local trader	None
13	*Triplochiton scleroxylon*	Local trader	None
**Treated wood samples**			
**Nr.**	**Sample**	**Source**	**Treatment**
14	*Acer ssp.*	Local trader	Photodegraded for 168 hours with an OSRAM Ultra Vitalux 300 W lamp. Distance: 40 cm
15	*Fagus sylvatica*	Local trader	Degraded by *Trametes versicolor* white rot fungus, mass loss: 11 %
16	*Picea abies*	Field sample, Kaunertal, Tyrol. Provided by the microbiological institute, University of Innsbruck, Austria	Slightly degraded by *Serpula lacrimans* brown rot fungus
17	*Picea abies*	Field sample, Kaunertal, Tyrol	Degraded by *Serpula lacrimans* brown rot fungus

Polysaccharides and lignin samples			
**Nr.**	**Sample**	**Source**	**Treatment**
18	Bacterial cellulose	CelluloseLab, Canada<br>www.celluloselab.com	None
19	Cellulose for column chromatography	Carl Roth GmbH + Co KG<br>Karlsruhe, Germany	None
20	Chitin from shrimp shells	Carl Roth GmbH + Co KG<br>Karlsruhe, Germany	None
21	Cotton nettle	Bau-forensik.eu	None
22	Fluffy mycelium of *Serpula lacrymans*	Field sample, Kaunertal, Tyrol. Provided by the microbiological institute, University of Innsbruck, Austria	None
23	D(+)Xylose	Carl Roth GmbH + Co KG<br>Karlsruhe, Germany	
24	Filter paper	Macherey-Nagel GmbH & Co KG<br>Düren, Germany	Stored at 2-3 % relative humidity
25	Microcrystalline cellulose, 50 µm	Serva Electrophoresis GmbH<br>Heidelberg, Germany	Stored at 11 % relative humidity until equilibrium
26	Microcrystalline cellulose, 50 µm		Stored at 44 % relative humidity until equilibrium
27	Microcrystalline cellulose, 50 µm		Stored at 85 % relative humidity until equilibrium
28	*Picea abies*	Local trader	Artificially weathered: 1000 hours of exposure to Xenon lamp, with cyclic water bath
29	Organosolv lignin from Eucalyptus ssp.	Fraunhofer Center for Chemical-biotechnological Processes CBP<br>Leuna, Germany	Dissolved in 60 % ethanol. Concentration: 0.1 %

Table S2: Peak Positions of the clusteroluminogens

Nr.	Literature	Class	Sample name	Measurement conditions	Ex [nm]	Em [nm]	Stokes-Shift [nm]	Residuals [nm]	Ex [eV]	Em [eV]	Stokes Shift [eV]
	**Polysaccharides and lignin measured in this study**										
01	this study	Wood (untreated)	Abies alba	solid	430	536	106	-10.5	2.884	2.313	0.570
02	this study	Wood (untreated)	Acer spp.	solid	395	475	80	6.0	3.139	2.611	0.529
03	this study	Wood (untreated)	Alnus glutinosa	solid	380	470	90	-4.8	3.263	2.638	0.625
04	this study	Wood (untreated)	Betula pendula	solid	390	478	88	-1.5	3.179	2.594	0.585
05	this study	Wood (untreated)	Carpinus betulus	solid	380	464	84	0.3	3.263	2.672	0.591
06	this study	Wood (untreated)	Fagus sylvatica	solid	390	484	94	-6.6	3.179	2.562	0.618
07	this study	Wood (untreated)	Fraxinus exselsior	solid	390	478	88	-1.5	3.179	2.594	0.585
08	this study	Wood (untreated)	Ochroma spp.	solid	355	432	77	2.3	3.493	2.870	0.623
09	this study	Wood (untreated)	Picea abies	solid	430	540	110	-13.9	2.884	2.296	0.587
10	this study	Wood (untreated)	Prunus avium sapwood	solid	365	454	89	-6.3	3.397	2.731	0.666
11	this study	Wood (untreated)	Terminalia superba	solid	415	510	95	-3.5	2.988	2.431	0.557
12	this study	Wood (untreated)	Tilia spp.	solid	410	516	106	-13.6	3.024	2.403	0.621
13	this study	Wood (untreated)	Triplochiton scleroxylon	solid	420	524	104	-10.4	2.952	2.366	0.586
14	this study	Wood (modified)	Acer spp. (photodegraded)	solid	440	565	125	-25.0	2.818	2.195	0.623
14	this study	Wood (modified)	Acer spp. (photodegraded)	solid	495	585	90	13.1	2.505	2.120	0.385
15	this study	Wood (modified)	Fagus sylvatica (degraded by T. versicolor)	solid	440	550	110	-12.3	2.818	2.255	0.564
15	this study	Wood (modified)	Fagus sylvatica (degraded by T. versicolor)	solid	490	580	90	12.4	2.531	2.138	0.393
16	this study	Wood (modified)	Picea abies (slightly degraded by S. lacrymans)	solid	435	545	110	-13.1	2.851	2.275	0.575
16	this study	Wood (modified)	Picea abies (slightly degraded by S. lacrymans)	solid	495	585	90	13.1	2.505	2.120	0.385
17	this study	Wood (modified)	Picea abies (degraded by S. lacrymans)	solid	440	560	120	-20.7	2.818	2.214	0.604
17	this study	Wood (modified)	Picea abies (degraded by S. lacrymans)	solid	495	590	95	8.9	2.505	2.102	0.403
18	this study	Polysaccharides	bacterial Cellulose (dry)	solid	315	384	69	2.8	3.937	3.229	0.707
19	this study	Polysaccharides	Cellulose for column chromatography	powder in cuvette	325	388	63	9.5	3.815	3.196	0.620
20	this study	Polysaccharides	Chitin from crab shells	powder in cuvette	335	420	85	-7.5	3.701	2.952	0.749
21	this study	Polysaccharides	Cotton nettle	solid	335	420	85	-7.5	3.701	2.952	0.749
21	this study	Polysaccharides	Cotton nettle	solid	360	455	95	-12.1	3.444	2.725	0.719
22	this study	Polysaccharides	Fluffy mycelium of S. lacrymans	solid	375	480	105	-18.2	3.307	2.583	0.723
23	this study	Saccharides	D(+)Xylose	powder in cuvette	325	402	77	-2.4	3.815	3.085	0.731
24	this study	Polysaccharides	Filter paper dry (stored at 2-3% humidity)	solid	360	455	95	-12.1	3.444	2.725	0.719
25	this study	Polysaccharides	Microcrystalline cellulose (stored at 11% humidity)	powder in cuvette	330	420	90	-12.5	3.758	2.952	0.805
25	this study	Polysaccharides	Microcrystalline cellulose (stored at 11% humidity)	powder in cuvette	360	455	95	-12.1	3.444	2.725	0.719
26	this study	Polysaccharides	Microcrystalline cellulose (stored at 44% humidity)	powder in cuvette	325	420	95	-17.5	3.815	2.952	0.863
26	this study	Polysaccharides	Microcrystalline cellulose (stored at 44% humidity)	powder in cuvette	360	455	95	-12.1	3.444	2.725	0.719
27	this study	Polysaccharides	Microcrystalline cellulose (stored at 85% humidity)	powder in cuvette	320	425	105	-26.8	3.875	2.918	0.957
27	this study	Polysaccharides	Microcrystalline cellulose (stored at 85% humidity)	powder in cuvette	360	460	100	-16.3	3.444	2.696	0.749
28	this study	Polysaccharides	Picea abies (artificially weathered)	solid	400	515	115	-22.8	3.100	2.408	0.692
29	this study	Lignin	Organosolv-Lignin Eucalyptus (0.1 m-% in 60%igem EthOH)	frontface in quartz cuvette	310	394	84	-10.6	4.000	3.147	0.853

Nr.	Literature	Class	Sample name	Measurement conditions	Ex [nm]	Em [nm]	Stokes-Shift [nm]	Residuals [nm]	Ex [eV]	Em [eV]	Stokes Shift [eV]
	**Polysaccharides and lignin: Literature spectra**										
[35]	Auxenfans et al (2017)	Lignocellulose	miscanthus x giganteus, untreated	solid in KBr pellets	366	436	70	9.9	3.388	2.844	0.544
[35]	Auxenfans et al (2017)	Lignocellulose	miscanthus x giganteus, steam exploded (combined severity factor = 2.8)		354	421	67	10.6	3.503	2.945	0.557
[35]	Auxenfans et al (2017)	Lignocellulose	populus spp., untreated		376	445	69	12.3	3.298	2.787	0.511
[35]	Auxenfans et al (2017)	Lignocellulose	populus spp., steam exploded (combined severity factor = 2.7)		358	418	60	17.1	3.464	2.967	0.497
[35]	Auxenfans et al (2017)	Lignocellulose	Wheat straw, untreated		358	441	83	-2.3	3.464	2.812	0.652
[35]	Auxenfans et al (2017)	Lignocellulose	wheat straw, steam exploded (combined severity factor = 2.8)		352	417	65	12.0	3.523	2.974	0.549
[36]	Dramicanin et al (2018)	Saccharides	honey	front-face	345	435	90	-10.2	3.594	2.851	0.744
[36]	Dramicanin et al (2018)	Saccharides	fake honey (sucrose sirup stored in by bees during winter feed)	front-face	350	435	85	-5.2	3.543	2.851	0.692
[37]	Geng et al (2015)	Polysaccharides	Chitosan aerogel, crosslinked by glutaric dialdehyde	aerogel	337	401	64	10.5	3.680	3.092	0.587
[39]	Hoque et al (2023)	Lignocellulose	Unbleached Kraft Pulp	solid	410	536	126	-30.5	3.024	2.313	0.711
[39]	Hoque et al (2023)	Lignocellulose	waste unbleached pulp fibre	solid	325	420	95	-17.5	3.815	2.952	0.863
[39]	Hoque et al (2023)	Lignocellulose	fibre cement (commercial)	solid	360	450	90	-7.9	3.444	2.756	0.689
[39]	Hoque et al (2023)	Lignocellulose	Ligno-xylan (4-O-Methyl-D-glucurono-D-xylan)	solid	425	535	110	-14.6	2.918	2.318	0.600
[39]	Hoque et al (2023)	Lignocellulose	Lignocellulose nanofibrils	solid	410	524	114	-20.4	3.024	2.366	0.658
[39]	Hoque et al (2023)	Polysaccharides	alpha-cellulose		325	440	115	-34.4	3.815	2.818	0.997
[40]	Huang et al (2020)	Polysaccharides	Carboxymethyl chitosan		320	405	85	-9.9	3.875	3.062	0.813
[40]	Huang et al (2020)	Polysaccharides	Carboxymethyl chitosan (zinc doped)		360	436.8	76.8	3.3	3.444	2.839	0.606
[41]	Jiang et al (2021)	Polysaccharides	Microcrystalline cellulose	solid	312	410	98	-22.1	3.974	3.024	0.950
[41]	Jiang et al (2021)	Polysaccharides	Microcrystalline cellulose, NaOH-treated	solid	312	415	103	-26.3	3.974	2.988	0.986
[42]	Li et al (2019)	Polysaccharides	Carboxymethylated Nanocellulose	dispersion in water	360	450	90	-7.9	3.444	2.756	0.689
[43]	Li et al (2023)	Polysaccharides	aminoacid-modified cyclodextrin CDGly	aqueous solution 1.0 mM	350	440	90	-9.4	3.543	2.818	0.725
[43]	Li et al (2023)	Polysaccharides	aminoacid-modified cyclodextrin CDIle	aqueous solution 1.0 mM	330	415	85	-8.3	3.758	2.988	0.770
[43]	Li et al (2023)	Polysaccharides	aminoacid-modified cyclodextrin CDMet	aqueous solution 1.0 mM	345	435	90	-10.2	3.594	2.851	0.744
[43]	Li et al (2023)	Polysaccharides	aminoacid-modified cyclodextrin CDCys	aqueous solution 1.0 mM	370	445	75	6.3	3.351	2.787	0.565
[43]	Li et al (2023)	Polysaccharides	aminoacid-modified cyclodextrin CDGlu	aqueous solution 1.0 mM	340	425	85	-6.8	3.647	2.918	0.729
[43]	Li et al (2023)	Polysaccharides	aminoacid-modified cyclodextrin CDGln	aqueous solution 1.0 mM	340	430	90	-11.0	3.647	2.884	0.763
[43]	Li et al (2023)	Polysaccharides	aminoacid-modified cyclodextrin CDAsn	aqueous solution 1.0 mM	325	400	75	-0.7	3.815	3.100	0.715
[43]	Li et al (2023)	Polysaccharides	aminoacid-modified cyclodextrin CDArg	aqueous solution 1.0 mM	325	410	85	-9.1	3.815	3.024	0.791
[43]	Li et al (2023)	Polysaccharides	aminoacid-modified cyclodextrin CDPhe	aqueous solution 1.0 mM	375	445	70	11.3	3.307	2.787	0.520
[43]	Li et al (2023)	Polysaccharides	aminoacid-modified cyclodextrin CDTrp	aqueous solution 1.0 mM	375	480	105	-18.2	3.307	2.583	0.723
[43]	Li et al (2023)	Polysaccharides	aminoacid-modified cyclodextrin CDHis	aqueous solution 1.0 mM	390	480	90	-3.2	3.179	2.583	0.596
[44]	Nakamura et al (2014)	Polysaccharides	Ramie textile	solid	375	450	75	7.1	3.307	2.756	0.551
[35]	Auxenfans et al (2017)	Lignin	Dehydrogenative polymer (DHP) from 100 % coniferyl alcohol	solid in KBr pellet	372	438	66	14.3	3.333	2.831	0.502
[35]	Auxenfans et al (2017)	Lignin	Dehydrogenative polymer (DHP) from 50/50 % coniferyl alcohol and sinapyl alcohol	solid in KBr pellet	384	450	66	16.1	3.229	2.756	0.474
[45]	Radotic et al (2006)	Lignin	Dehydrogenative polymer (DHP) as lignin model substance	dispersion in water	365	440	75	5.6	3.397	2.818	0.579
[45]	Radotic et al (2006)	Lignin	Dehydrogenative polymer (DHP) as lignin model substance	solution in choloform/ methanol 3:1	360	430	70	9.0	3.444	2.884	0.561

Nr.	Literature	Class	Sample name	Measurement conditions	Ex [nm]	Em [nm]	Stokes-Shift [nm]	Residuals [nm]	Ex [eV]	Em [eV]	Stokes Shift [eV]
			**Other Clusteroluminogens**								
[44]	Nakamura et al (2014)	Polypeptides	Silk (fibroin and sericin)	solid	363	434	71	8.6	3.416	2.857	0.559
[46]	Bao et al (2023)	Polypeptides	Bovine Serum Albumin	aqueous solution (20 mg/ml)	365	445	80	1.3	3.397	2.787	0.611
[47]	Chen et al (2018)	Polypeptides	L-Lysin 0.1 M	solution	365	445	80	1.3	3.397	2.787	0.611
[47]	Chen et al (2018)	Polypeptides	L-Lysin 0.1 M	solution	380	490	110	-21.6	3.263	2.531	0.733
[47]	Chen et al (2018)	Polypeptides	Epsilon-Poly-L-Lysine	solution in water	336	407	71	4.4	3.690	3.047	0.644
[48]	Komura et al (2021)	Polypeptides	advanced glycation endproducts (AGEs) of	solution	333	420	87	-9.5	3.724	2.952	0.771
[49]	Millington (2020)	Polypeptide	white wool (keratin)	solid	365	430	65	14.0	3.397	2.884	0.514
[49]	Millington (2020)	Polypeptides	white rabbit hair (keratin)	solid	365	440	75	5.6	3.397	2.818	0.579
[49]	Millington (2020)	Polypeptides	white cashmere (keratin)	solid	365	440	75	5.6	3.397	2.818	0.579
[49]	Millington (2020)	Polypeptides	white mink hair (keratin)	solid	375	455	80	2.9	3.307	2.725	0.581
[49]	Millington (2020)	Polypeptides	white human hair (keratin)	solid	380	465	85	-0.5	3.263	2.667	0.596
[49]	Millington (2020)	Polypeptides	casein	solid	365	440	75	5.6	3.397	2.818	0.579
[49]	Millington (2020)	Polypeptides	Bovine Serum Albumin	solid	375	450	75	7.1	3.307	2.756	0.551
[49]	Millington (2020)	Polypeptides	Papain	solid	355	420	65	12.5	3.493	2.952	0.541
[49]	Millington (2020)	Polypeptides	Bovine Collagen	solid	355	425	70	8.2	3.493	2.918	0.575
[49]	Millington (2020)	Polypeptides	White cacadoo feather (beta-Keratin)	solid	355	430	75	4.0	3.493	2.884	0.609
[50]	Séro et al (2013)	Polypeptides	advanced glycation endproducts (AGEs) of bovine serum albumin (BSA)		340	410	70	5.9	3.647	3.024	0.623
[51]	Xu et al (2020)	Polypeptides	Gelatin	solution	320	395	75	-1.4	3.875	3.139	0.736
[51]	Xu et al (2020)	Polypeptides	Gelatin	solid	380	470	90	-4.8	3.263	2.638	0.625
[52]	Zhao et al (2010)	Polypeptides	Human thumbnail		375	460	85	-1.3	3.307	2.696	0.611
[53]	Zhao et al (2024)	Polypeptides	poly(gamma-benzyl-glutamate)	solution	340	400	60	14.3	3.647	3.100	0.547
[54]	Xie et al (2023)	Organic small molecules	methyl 3-aminocrotonate (MAC)	solution (ethanol)	351	430	79	0.0	3.533	2.884	0.649
[55]	Zhang et al (2017)	Organic small molecules	hexanal oxime	solid	372	443	71	10.0	3.333	2.799	0.534
[56]	Chu et al (2022)	synthetic polymers	aliphatic polyester P1	solution 10-3 M in DCM	350	420	70	7.5	3.543	2.952	0.590
[56]	Chu et al (2022)	synthetic polymers	aliphatic polyester P2	solid	380	451	71	11.3	3.263	2.749	0.514
[56]	Chu et al (2022)	synthetic polymers	aliphatic polyester P3	solution 10-3 M in DCM	385	460	75	8.7	3.221	2.696	0.525
[56]	Chu et al (2022)	synthetic polymers	aliphatic polyester P3	solid	380	449	69	13.0	3.263	2.762	0.501
[56]	Chu et al (2022)	synthetic polymers	aliphatic polyester P4	solution 10-3 M in DCM	380	460	80	3.7	3.263	2.696	0.568
[56]	Chu et al (2022)	synthetic polymers	aliphatic polyester P4	solid	380	453	73	9.6	3.263	2.737	0.526
[56]	Chu et al (2022)	synthetic polymers	aliphatic polyester P5	solution 10-3 M in DCM	380	460	80	3.7	3.263	2.696	0.568
[56]	Chu et al (2022)	synthetic polymers	aliphatic polyester P5	solid	380	454	74	8.7	3.263	2.731	0.532
[56]	Chu et al (2022)	synthetic polymers	aliphatic polyester P6	solution 10-3 M in DCM	380	462	82	2.0	3.263	2.684	0.579
[56]	Chu et al (2022)	synthetic polymers	aliphatic polyester P6	solid	380	453	73	9.6	3.263	2.737	0.526
[56]	Chu et al (2022)	synthetic polymers	aliphatic polyester P9	solution 10-3 M in DCM	385	477	92	-5.7	3.221	2.600	0.621
[56]	Chu et al (2022)	synthetic polymers	aliphatic polyester P10	solution 10-3 M in DCM	380	474	94	-8.1	3.263	2.616	0.647
[56]	Chu et al (2022)	synthetic polymers	aliphatic polyester P11	solution 10-3 M in DCM	390	477	87	-0.7	3.179	2.600	0.580
[56]	Chu et al (2022)	synthetic polymers	aliphatic polyester P12	solution 10-3 M in DCM	385	475	90	-4.0	3.221	2.611	0.610
[56]	Chu et al (2022)	synthetic polymers	aliphatic polyester P13	solution 10-3 M in DCM	390	477	87	-0.7	3.179	2.600	0.580
[56]	Chu et al (2022)	synthetic polymers	aliphatic polyester P13	solid	470	533	63	32.0	2.638	2.326	0.312
[56]	Chu et al (2022)	synthetic polymers	aliphatic polyester P14	solution 10-3 M in DCM	360	445	85	-3.7	3.444	2.787	0.658
[56]	Chu et al (2022)	synthetic polymers	aliphatic polyester P14	solid	400	471	71	14.4	3.100	2.633	0.467
[56]	Chu et al (2022)	synthetic polymers	aliphatic polyester P15	solution 10-3 M in DCM	400	490	90	-1.6	3.100	2.531	0.569
[56]	Chu et al (2022)	synthetic polymers	aliphatic polyester P15	solution 10-3 M in DCM	470	540	70	26.1	2.638	2.296	0.342

Nr.	Literature	Class	Sample name	Measurement conditions	Ex [nm]	Em [nm]	Stokes-Shift [nm]	Residuals [nm]	Ex [eV]	Em [eV]	Stokes Shift [eV]
[56]	Chu et al (2022)	synthetic polymers	aliphatic polyester P16	solution 10-3 M in DCM	380	467	87	-2.2	3.263	2.655	0.608
[56]	Chu et al (2022)	synthetic polymers	aliphatic polyester P16	solid	460	532	72	22.9	2.696	2.331	0.365
[56]	Chu et al (2022)	synthetic polymers	aliphatic polyester P17	solution 10-3 M in DCM	390	475	85	1.0	3.179	2.611	0.569
[56]	Chu et al (2022)	synthetic polymers	aliphatic polyester P17	solid	470	536	66	29.5	2.638	2.313	0.325
[56]	Chu et al (2022)	synthetic polymers	aliphatic polyester P18	solution 10-3 M in DCM	390	480	90	-3.2	3.179	2.583	0.596
[56]	Chu et al (2022)	synthetic polymers	aliphatic polyester P18	solid	410	485	75	12.6	3.024	2.557	0.468
[56]	Chu et al (2022)	synthetic polymers	aliphatic polyester P19	solution 10-5 to 10-1 M in DCM	410	500	90	-0.1	3.024	2.480	0.544
[56]	Chu et al (2022)	synthetic polymers	aliphatic polyester P19	solid	480	572	92	9.1	2.583	2.168	0.416
[56]	Chu et al (2022)	synthetic polymers	aliphatic polyester P20	solution 10-5 to 10-1 M in DCM	400	502	102	-11.8	3.100	2.470	0.630
[56]	Chu et al (2022)	synthetic polymers	aliphatic polyester P20	solid	480	571	91	10.0	2.583	2.172	0.412
[56]	Chu et al (2022)	synthetic polymers	aliphatic polyester P21	solution 10-5 to 10-1 M in DCM	400	499	99	-9.2	3.100	2.485	0.615
[56]	Chu et al (2022)	synthetic polymers	aliphatic polyester P21	solid	470	568	98	2.5	2.638	2.183	0.455
[56]	Chu et al (2022)	synthetic polymers	aliphatic polyester P22	solution 10-5 to 10-1 M in DCM	410	501	91	-0.9	3.024	2.475	0.549
[56]	Chu et al (2022)	synthetic polymers	aliphatic polyester P22	solution 10-3 M in DCM	480	571	91	10.0	2.583	2.172	0.412
[56]	Chu et al (2022)	synthetic polymers	aliphatic polyester P23	solution 10-5 to 10-1 M in DCM	410	505	95	-4.3	3.024	2.455	0.569
[56]	Chu et al (2022)	synthetic polymers	aliphatic polyester P23	solid	480	571	91	10.0	2.583	2.172	0.412
[56]	Chu et al (2022)	synthetic polymers	aliphatic polyester P24	solution 10-5 to 10-1 M in DCM	410	501	91	-0.9	3.024	2.475	0.549
[56]	Chu et al (2022)	synthetic polymers	aliphatic polyester P24	solid	480	567	87	13.3	2.583	2.187	0.396
[57]	Miao et al (2016)	synthetic polymers	hyperbranched polyether epoxy (EHBPE)	solution in ethanol	365	465	100	-15.5	3.397	2.667	0.731
[58]	Shang et al (2017)	synthetic polymers	poly[(maleic anhydride-alt-vinyl pyrrolidone (PMVP) - low DP	solution (DMSO)	375	443	68	13.0	3.307	2.799	0.508
[58]	Shang et al (2017)	synthetic polymers	poly[(maleic anhydride-alt-vinyl pyrrolidone (PMVP) - high DP	solution (DMSO)	456	546	90	7.1	2.719	2.271	0.448
[58]	Shang et al (2017)	synthetic polymers	poly[(maleic anhydride-alt-vinyl pyrrolidone (PMVP) - low DP	solid	371	450	79	3.1	3.342	2.756	0.587
[58]	Shang et al (2017)	synthetic polymers	poly[(maleic anhydride-alt-vinyl pyrrolidone (PMVP) - high DP	solid	463	545	82	14.9	2.678	2.275	0.403
[59]	Wang et al (2021)	synthetic polymers	poly(vinyl alcohol) PVA-105		360	428	68	10.7	3.444	2.897	0.547
[59]	Wang et al (2021)	synthetic polymers	poly(vinyl alcohol) PVA-224		340	410	70	5.9	3.647	3.024	0.623
[60]	Zhang et al (2010)	synthetic polymers	hyperbranched poly(ester-amide-ether)-mono		330	395	65	8.6	3.758	3.139	0.618
[60]	Zhang et al (2010)	synthetic polymers	hyperbranched poly(ester-amide-ether)		330	400	70	4.3	3.758	3.100	0.658
[60]	Zhang et al (2010)	synthetic polymers	hyperbranched poly(ester-amide-ether)-di		330	395	65	8.6	3.758	3.139	0.618
[60]	Zhang et al (2010)	synthetic polymers	hyperbranched poly(ester-amide-ether)-tri		330	385	55	17.0	3.758	3.221	0.537
[61]	Zhou et al (2016)	synthetic polymers	Poly-Acrylonitrile (PAN)	solid	348	405	57	18.1	3.563	3.062	0.501
[62]	Bavya et al (2024)	Carbon Dots	cellulose carbon dots, doped with nitrogen (urea)	aqueous solution	352	439	87	-6.6	3.523	2.825	0.698
[63]	Wang et al (2013)	Carbon Dots	Carbon dots from overcooked BBQ		440	520	80	13.0	2.818	2.385	0.434
[64]	Zattar et al (2022)	Carbon Dots	aminated cellulose carbon dots	aqueous solution	340	395	55	18.6	3.647	3.139	0.508
[64]	Zattar et al (2022)	Carbon Dots	cellulose carbon dots, surface activated with PEG	aqueous solution	300	375	75	-4.6	4.133	3.307	0.827
[64]	Zattar et al (2022)	Carbon Dots	raw cellulose carbon dots	aqueous solution	360	445	85	-3.7	3.444	2.787	0.658
[65]	Zhu et al (2012)	Polymer dots	polyvinyl alcohol polymer dots	aqueous solution	375	475	100	-14.0	3.307	2.611	0.696
[65]	Zhu et al (2012)	Polymer dots	polyethylene imine polymer dots		362	465	103	-18.5	3.425	2.667	0.759
[66]	Zhu et al (2013)	Carbon Dots	carbon dots CD1 (condensation product of ethylene diamine + citric acid)		360	443	83	-2.0	3.444	2.799	0.645
[66]	Zhu et al (2013)	Carbon Dots	carbon dots CD3 (condensation product of ethylene diamine + citric acid)		380	450	70	12.1	3.263	2.756	0.508
[66]	Zhu et al (2013)	Carbon Dots	carbon dots CD4 (condensation product of ethylene diamine + citric acid)		380	445	65	16.3	3.263	2.787	0.477
[67]	Zhu et al (2014)	Polymer dots	polyethylene imine polymer dots (crosslinked by carbon tetrachloride)	solid	400	475	75	11.0	3.100	2.611	0.489

Table S3: Peak Positions of the conventional fluorophores

Nr.	Literature	Ex [nm]	Em [nm]	Stokes-Shift [nm]	Residuals [nm]	Ex [eV]	Em [eV]	Stokes Shift [eV]
	**Conventional fluorophores**							
[68]	from George McNamara. "PubSpectra - Open Data Access Fluorescence Spectra" (2012) Available at: http://works.bepress.com/gmcnamara/9/							
	Fluorophore (McNamara 2005 for Paddock 2.0)	Abs or	Emissio	Stokes shift				
	Toluene	220	290	70	-12.8			
	Phenol	220	292	72	-14.5			
	2-methylbenzoxazole	231	300	69	-10.2			
	Diphenyl-anthracene	279	302	23	36.1			
	Tyrosine (Tyr)	225	303	78	-18.8			
	Tyrosine (Tyr)	275	303	28	31.2			
	Naphthalene	220	322	102	-39.8			
	Biphenyl	247	326	79	-16.2			
	Ethyl-p-dimethylaminobenzoate	309	330	21	42.4			
	p-terphenyl	276	338	62	2.7			
	5-Hydroxytryptophan	277	339	62	2.8			
	3-BPEB	317	342	25	40.3			
	4-BPEB	315	344	29	36.6			
	Fluoroside H (terphenyl)	285	345	60	5.8			
	Stilbene	294	345	51	14.8			
	Tryptophan (Trp)	287	348	61	5.2			
	PPO	303	354	51	16.2			
	Fluoroside M (N,N-dimethylaniline)	260	357	97	-29.4			
	5-Fluorotryptophan	285	360	75	-6.9			
	p-quaterphenyl	293	363	70	-1.4			
	6-Fluorotryptophan	281	366	85	-16.0			
	linear thienocarbazole	269	372	103	-33.0			
	Fluoroside I (binaphthyl)	306	373	67	3.1			
	Diphenylbutadiene	330	373	43	27.1			
	Pyrene	241	381	140	-68.6			
	7-MeO-coumarin-AcOH abs	220	382	162	-90.5			
	Fluoroside Y (pyrene)	342	394	52	21.4			
	Anthracene	356	397	41	32.9			
	Indo-1	330	401	71	3.5			
	POPOP	256	407	151	-75.6			
	Fluoroside B (benzo[a]pyrene)	394	408	14	61.6			
	alpha-parinaric acid	303	410	107	-31.1			
	Cascade Blue	378	423	45	32.9			
	Diphenylhexatriene	353	425	72	6.2			
	7-amino-4-methylcoumarin	351	430	79	0.0			
	Fluoroside T (terthiophene)	358	432	74	5.3			
	Perylene	253	435	182	-102.2			
	EBFP	380	440	60	20.6			
	Fluoroside D (N,N-dimethylaminostilbene)	348	441	93	-12.3			
	Fluoroside E (perylene)	440	443	3	78.0			
	Coumarin 1	375	445	70	11.3			
	BFP(P4-3)	381	445	64	17.3			
	BFP(P4)	383	447	64	17.7			
	Pacific Blue	400	447	47	34.7			
	Quinine sulfate (in 0.05M H2SO4)	256	450	194	-111.9			
	6-chloro-7-hydroxy-coumarin-DCH	408	450	42	40.1			
	6-chloro-7-hydroxy-coumarin-3-carbamide	408	450	42	40.1			
	Quinine sulfate (in 0.5M H2SO4)	256	451	195	-112.7			
	Marina Blue	358	455	97	-14.1			
	PO-PRO-1	435	455	20	62.9			
	Fluoroside O (oxoperylene)	444	461	17	66.8			
	Coumarin 343	443	462	19	65.0			
	DAPI (in DMSO)	353	465	112	-27.5			
	9,10-Bis(phenylethynyl)anthracene	271	467	196	-111.2			
	Atto 425	426	470	44	41.2			
	Cycle 3 GFP (alphaGFP)	396	475	79	7.0			
	CFP W7	433	475	42	44.0			
	Coumarin 314	436	476	40	46.2			
	ECFP	434	477	43	43.3			
	Coumarin 30	406	478	72	14.5			
	4'-(2-benzo[b]furanyl)-3-hydroxychromone	413	481	68	18.9			
	BO-PRO-1	462	481	19	67.9			
	4'-diethylamino-3-hydroxyflavone	413	482	69	18.1			
	4'-Diethylamino-3-hydroxy-furano[3,2-g]flavone	418	482	64	23.1			
	Hoechst 33258 (in DMF)	354	486	132	-44.3			
	DAPI (in H2O)	344	487	143	-55.1			
	dansyl glycine (dioxane)	262	492	230	-141.3			

Nr.	Literature	Ex [nm]	Em [nm]	Stokes-Shift [nm]	Residuals [nm]	Ex [eV]	Em [eV]	Stokes Shift [eV]
	Acridine yellow	264	492	228	-139.3			
	C3-oxacyanine	485	497	12	77.4			
	Auramine O	431	499	68	21.8			
	Coumarin 6	456	500	44	45.9			
	Acrylodan	387	502	115	-24.8			
	BODIPY 494/505	495	504	9	81.5			
	Fura-2	335	505	170	-79.3			
	Cy2	489	506	17	73.8			
	Hoechst 33258 (in H2O)	345	507	162	-71.0			
	EGFP	488	507	19	72.0			
	DAMBO-T	498	507	9	82.0			
	GFP (Aequorea victoria wild type; avGFP)	395	508	113	-21.8			
	GFP (Aequorea victoria wild type; avGFP)	395	508	113	-21.8			
	CYA	491	508	17	74.2			
	YO-PRO-1	491	509	18	73.3			
	GFP (Renilla)	498	509	11	80.3			
	Vex-1 (violet GFP)	398	510	112	-20.5			
	ATTO-Dino 3 (dsDNA)	480	510	30	61.5			
	GFP Mut1	489	510	21	70.5			
	GFP S65T	489	510	21	70.5			
	GFP S65T	489	510	21	70.5			
	Proflavin (pH7)	261	511	250	-158.4			
	GFP (S65T)	489	511	22	69.6			
	DTAF (Dichlorotriazinylamino-fluorescein)	492	513	21	70.9			
	2',7;-Difluorofluorescein	490	514	24	68.1			
	Fluorescein	490	514	24	68.1			
	6-Carboxy-2',7'-difluorofluorescein	492	514	22	70.1			
	ATTO-Dino 4 (dsDNA)	485	515	30	62.2			
	DMAX-EP	492	515	23	69.2			
	DPAX-1-EP	494	515	21	71.2			
	5(6)-Carboxy-2',7'-difluorofluorescein	492	516	24	68.4			
	carboxyfluorescein	492	516	24	68.4			
	Calcein	494	516	22	70.4			
	Oregon Green 488	496	516	20	72.4			
	5-Carboxy-2',7'-difluorofluorescein	492	517	25	67.6			
	Alexa Fluor 488 (Alexa488)	495	519	24	68.9			
	Acridine orange	271	520	249	-156.0			
	3'6-Carboxyfluorescein (FAM)	495	520	25	68.0			
	5(6)-Carboxyfluorescein (FAM)	495	520	25	68.0			
	5-Carboxyfluorescein (FAM)	495	520	25	68.0			
	6-Carboxyfluorescein (FAM)	495	520	25	68.0			
	Rhodamine 110	496	520	24	69.0			
	Rhodol Green	496	523	27	66.5			
	Brilliant sulfoflavine, K salt	420	525	105	-11.2			
	3-hydroxyflavone	343	528	185	-90.7			
	NBD	459	529	70	24.4			
	Riboflavin	220	531	311	-216.3			
	ATTO-Dino 1 (dsDNA)	490	531	41	53.7			
	Lucifer Yellow CH, K salt	428	535	107	-11.6			
	Lucifer Yellow CH, Li salt	428	540	112	-15.9			
	Lucifer Yellow VS, Li salt	428	540	112	-15.9			
	C6-NBD-PC	475	540	65	31.1			
	ATTO-Mono 2 (dsDNA)	490	540	50	46.1			
	Lucifer Yellow CH	230	542	312	-215.6			
	BPTA-Tb3+-streptavidin	322	543	221	-124.4			
	BPTA-Tb3+	325	543	218	-121.4			
	NBD-amine (NBD-ethanolamine)	470	550	80	17.7			
	ATTO-Dino 3 (ssDNA)	480	570	90	10.8			
	ZnOEP	404	571	167	-66.0			
	ATTO-Dino 4 (ssDNA)	485	572	87	14.1			
	R-Phycoerythrin	480	578	98	4.1			
	Nile red	262	580	318	-215.6			
	MgOEP (in toluene)	410	582	172	-69.3			
	ATTO-Dino 1 (ssDNA)	490	582	92	10.7			
	Dapoxyl sulfonyl ethylenediamine	373	584	211	-108.0			
	di-4-AMSPPS	450	584	134	-31.0			
	4-Dimethylamino-4'-nitrostilbene	432	588	156	-52.4			
	2-CAREPBS	440	588	148	-44.4			
	di-6-ASP	462	595	133	-28.3			
	FM 1-43	479	598	119	-13.8			
	ATTO-Mono 2 (ssDNA)	490	600	110	-4.5			

Nr.	Literature	Ex [nm]	Em [nm]	Stokes-Shift [nm]	Residuals [nm]	Ex [eV]	Em [eV]	Stokes Shift [eV]
	di-4-ASPEC	468	602	134	-28.2			
	TTTA-Eu3+	336	615	279	-171.2			
	di-4-ASP	490	615	125	-17.2			
	BPTA-Eu3+-streptavidin	322	619	297	-188.6			
	BTTA-Eu3+	319	620	301	-192.4			
	BPTA-Eu3+	325	620	295	-186.4			
	dcm-pyran (in CH3CN)	458	624	166	-56.8			
	dcm-pyran (in MeOH)	464	624	160	-50.8			
	Tris(bpy)Ru	286	625	339	-229.6			
	Tetra-t-butylazaporphine	339	626	287	-177.5			
	ZnTMP	421	643	222	-109.8			
	ZnTPP	423	645	222	-109.5			
	TPP	419	649	230	-116.9			
	PtCP-antibody	380	650	270	-156.7			
	PtCP-Streptavidin	380	650	270	-156.7			
	PtPTP	380	650	270	-156.7			
	o-amino-TPP	406	654	248	-134.1			
	MgTPP	426	663	237	-121.7			
	MgTMP	427	664	237	-121.6			
	PtCP	402	675	273	-155.8			
	Porphin	396	683	287	-168.6			
	H4TPP	240	687	447	-328.0			
	Tetrakis(2,6-dichlorophenyl)porphyrin	419	716	297	-173.5			
	PdCP-Streptavidin	390	760	370	-239.6			
	PdPTP	390	760	370	-239.6			

Excitation-emission-matrices (EEMs) of untreated wood:

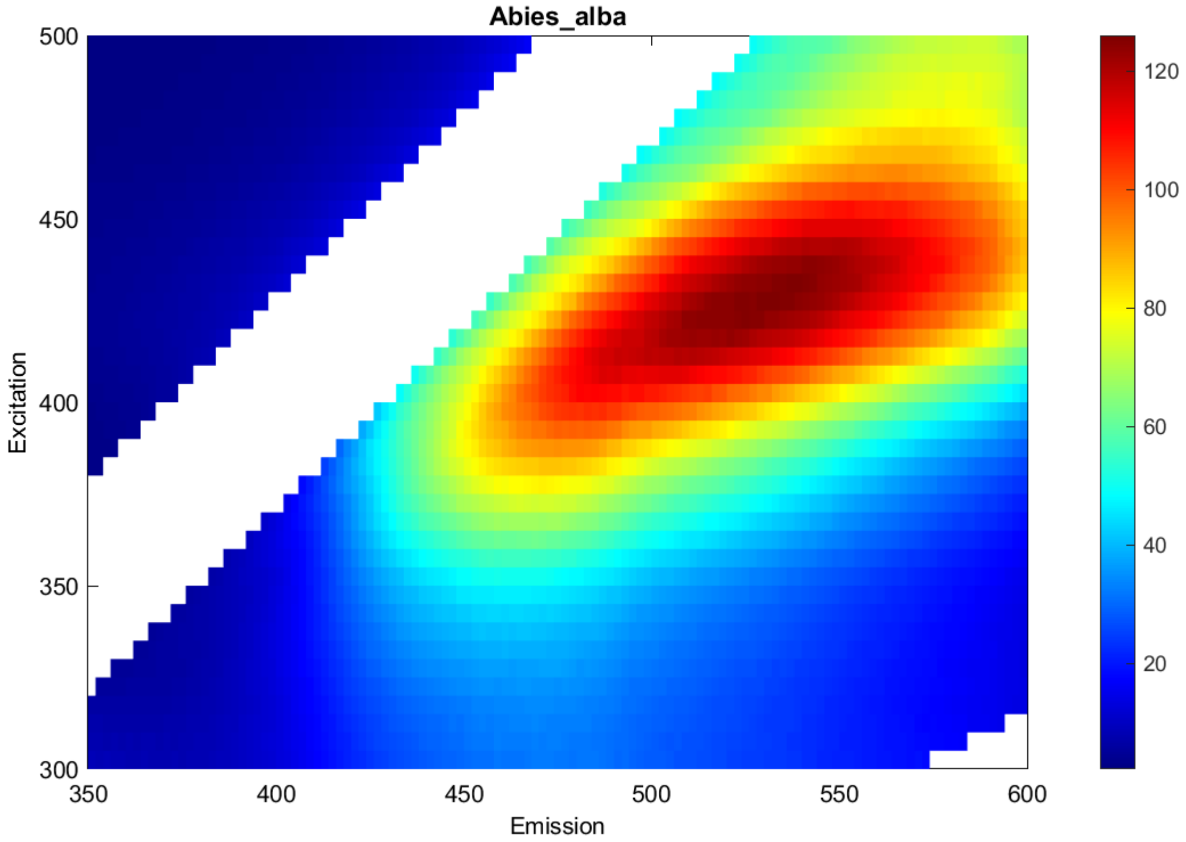

Figure S1: EEM of sample 1: untreated Abies alba wood

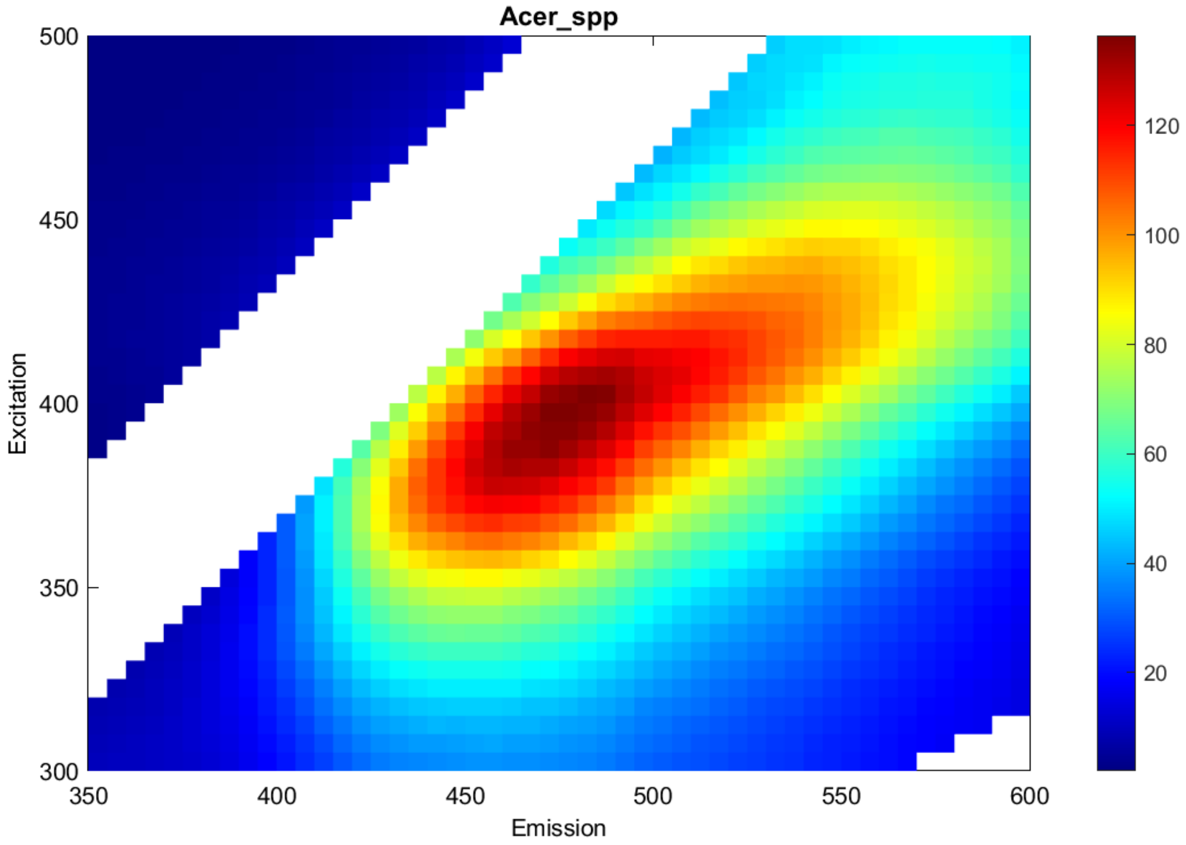

Figure S2: EEM of sample 2: untreated Acer ssp. wood

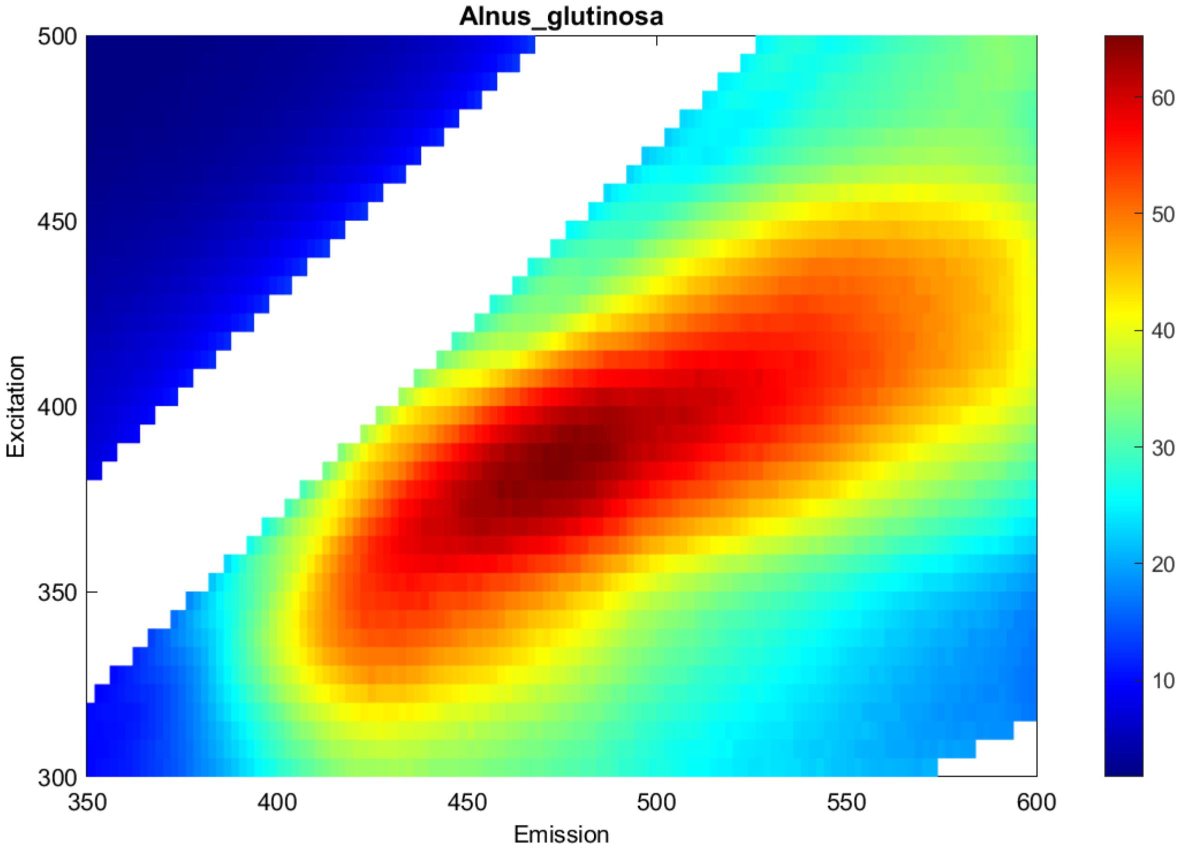

Figure S3: EEM of sample 3: untreated Alnus glutinosa wood

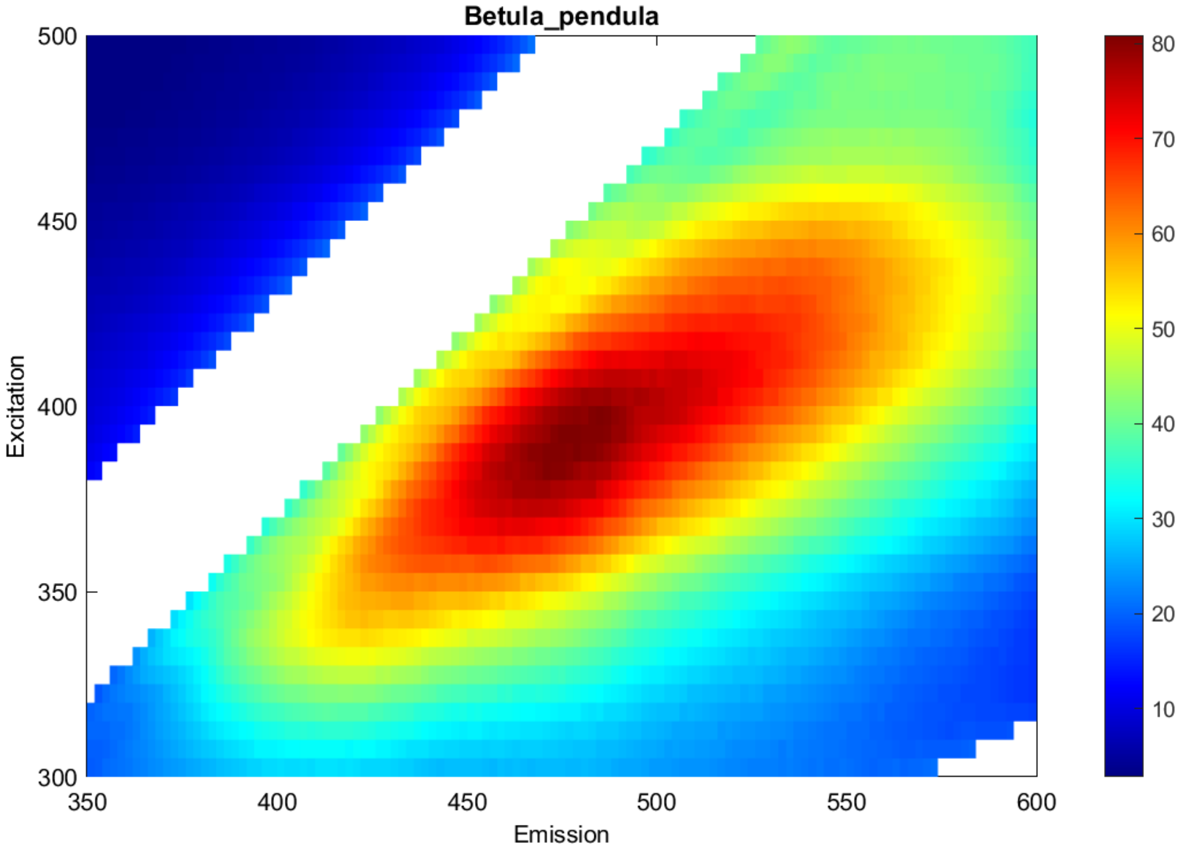

Figure S4: EEM of sample 4: untreated Betula pendula wood

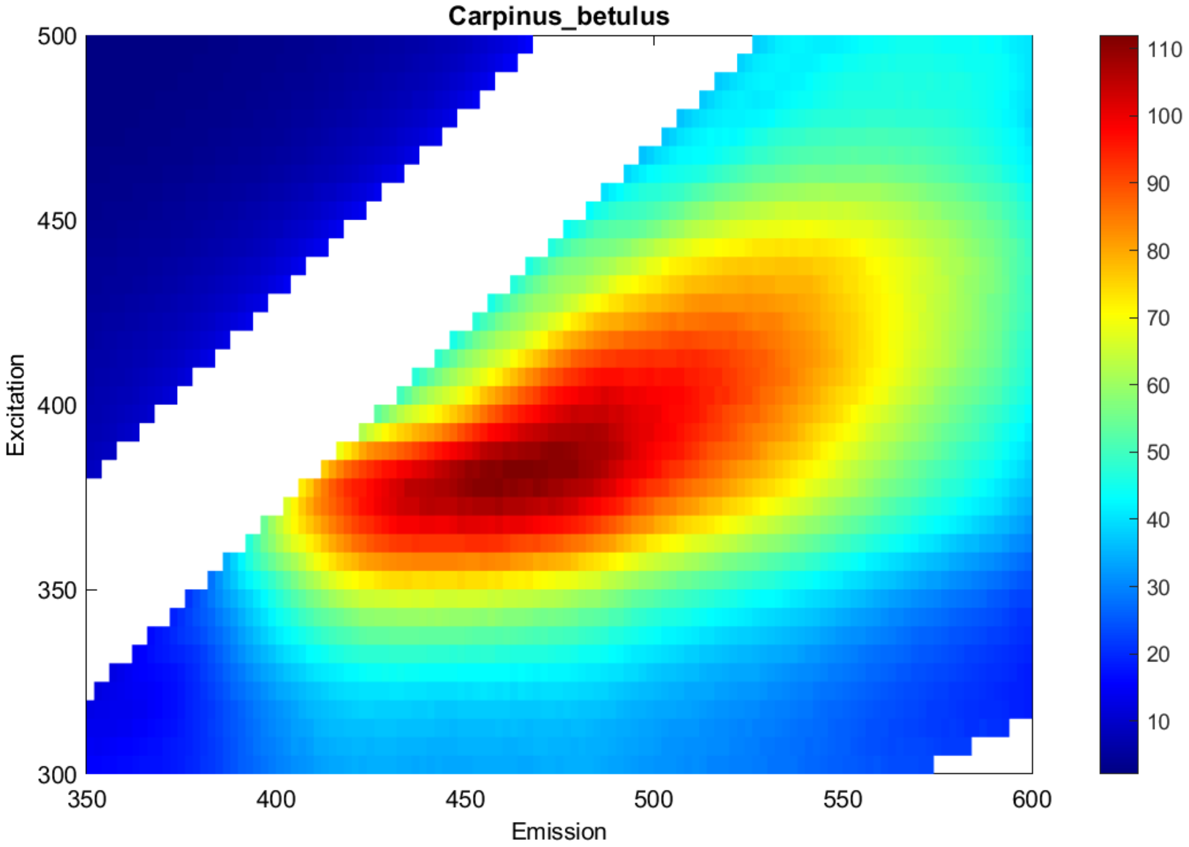

Figure S5: EEM of sample 5: untreated Carpinus betulus wood

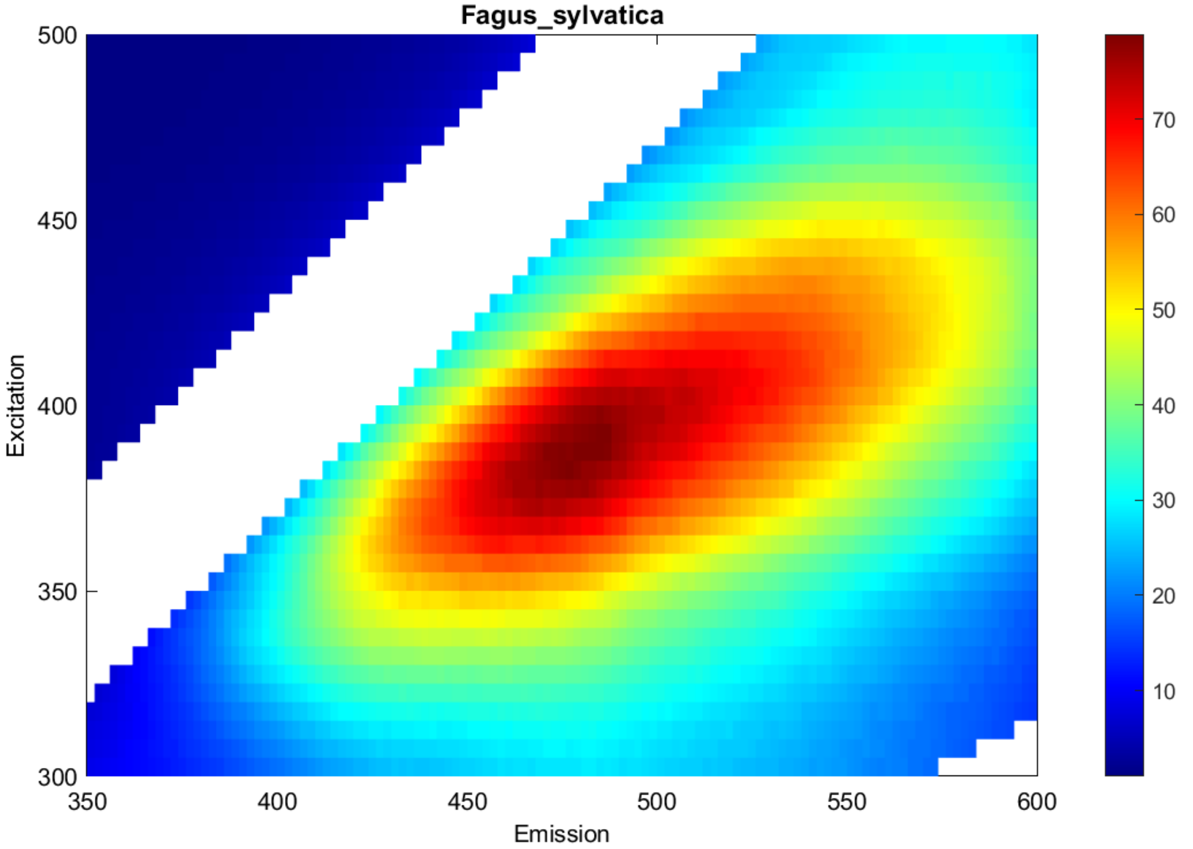

Figure S6: EEM of sample 6: untreated Fagus sylvatica wood

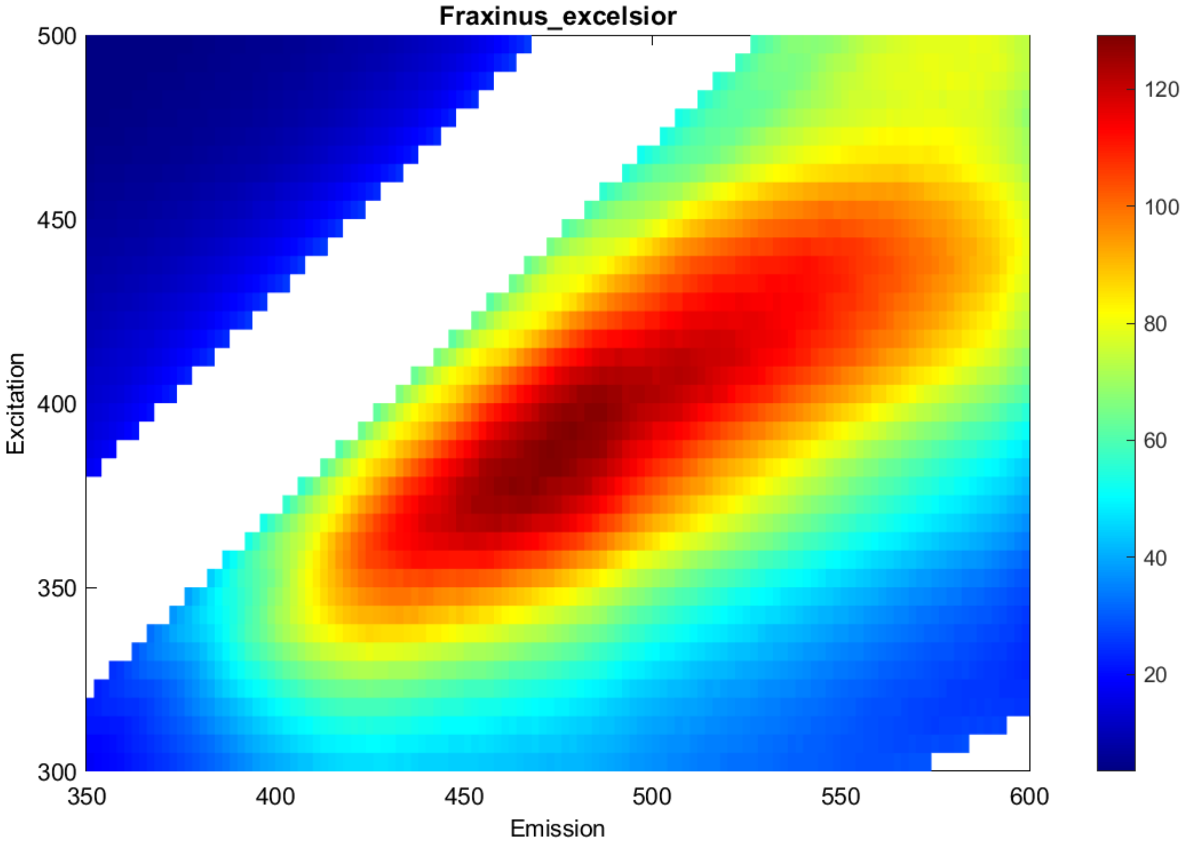

Figure S7: EEM of sample 7: untreated Fraxinus excelsior wood

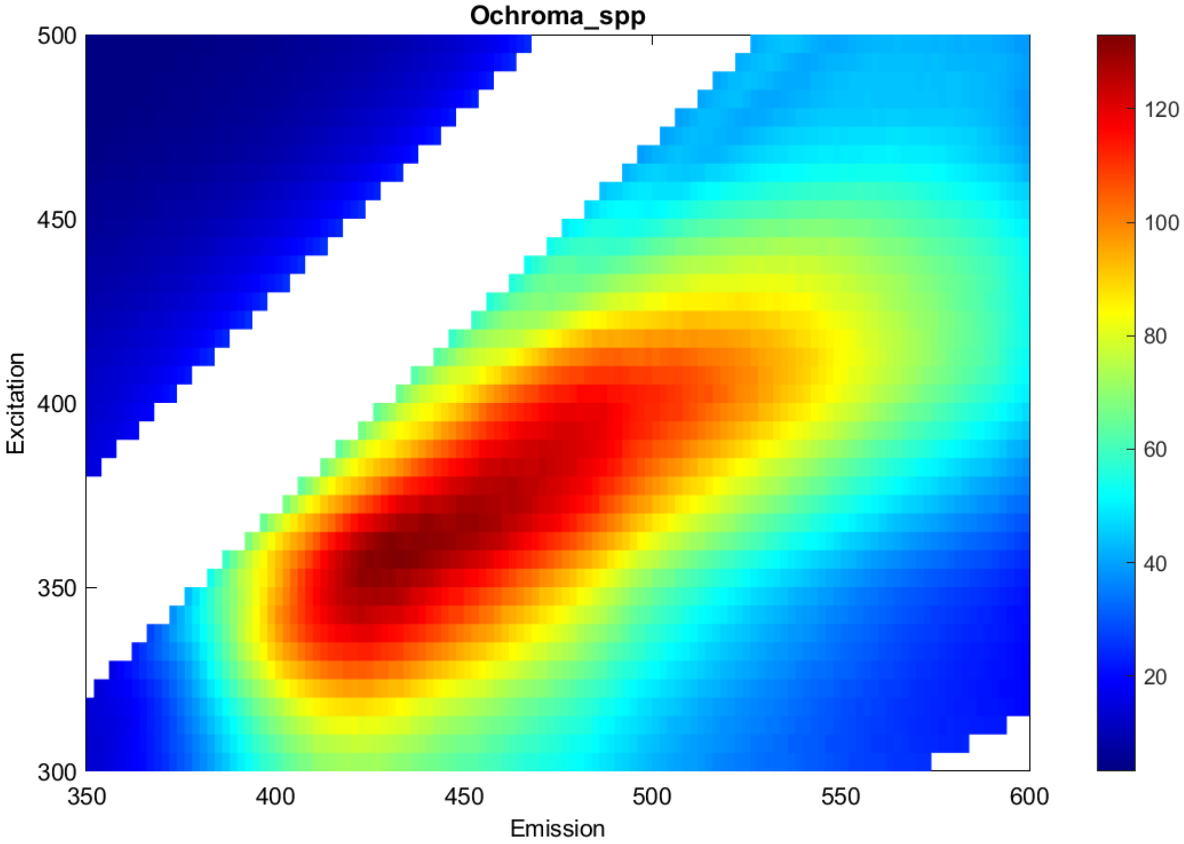

Figure S8: EEM of sample 8: untreated Ochroma ssp. wood

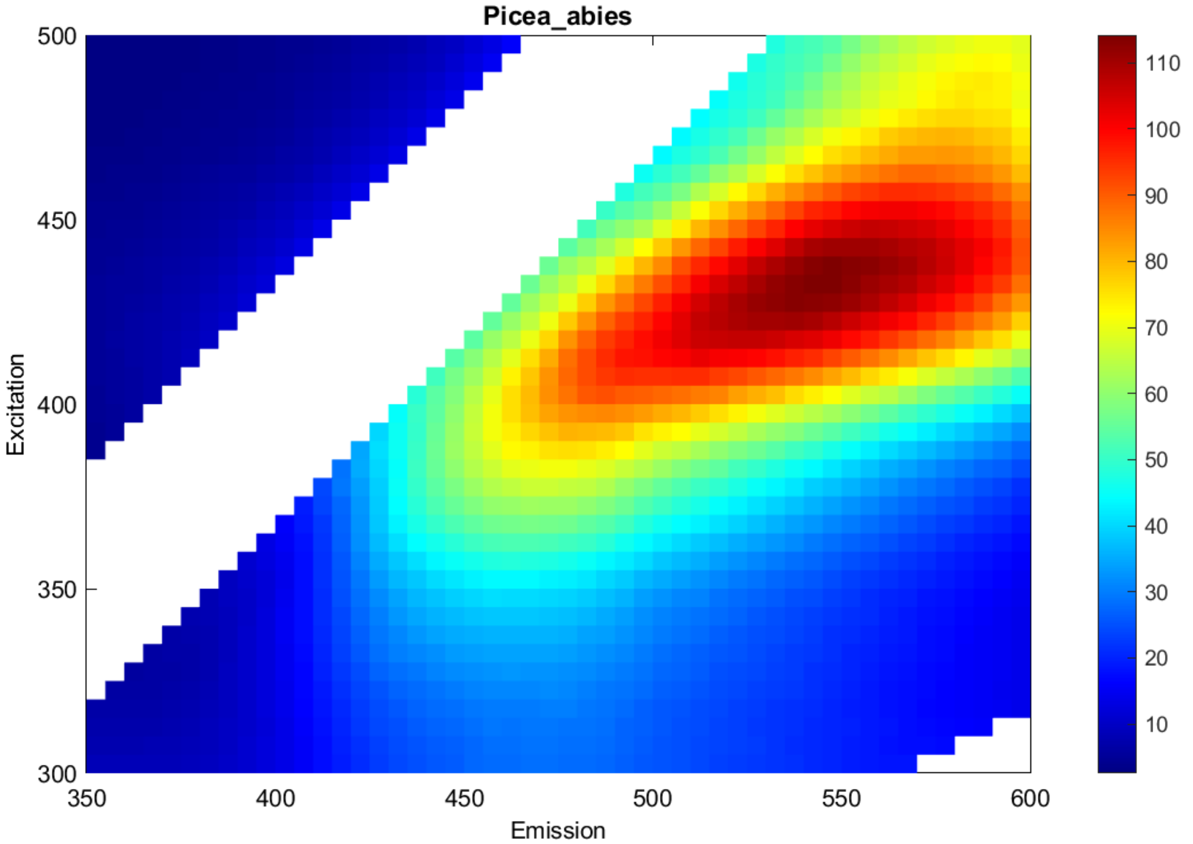

Figure S9: EEM of sample 9: untreated Picea abies wood

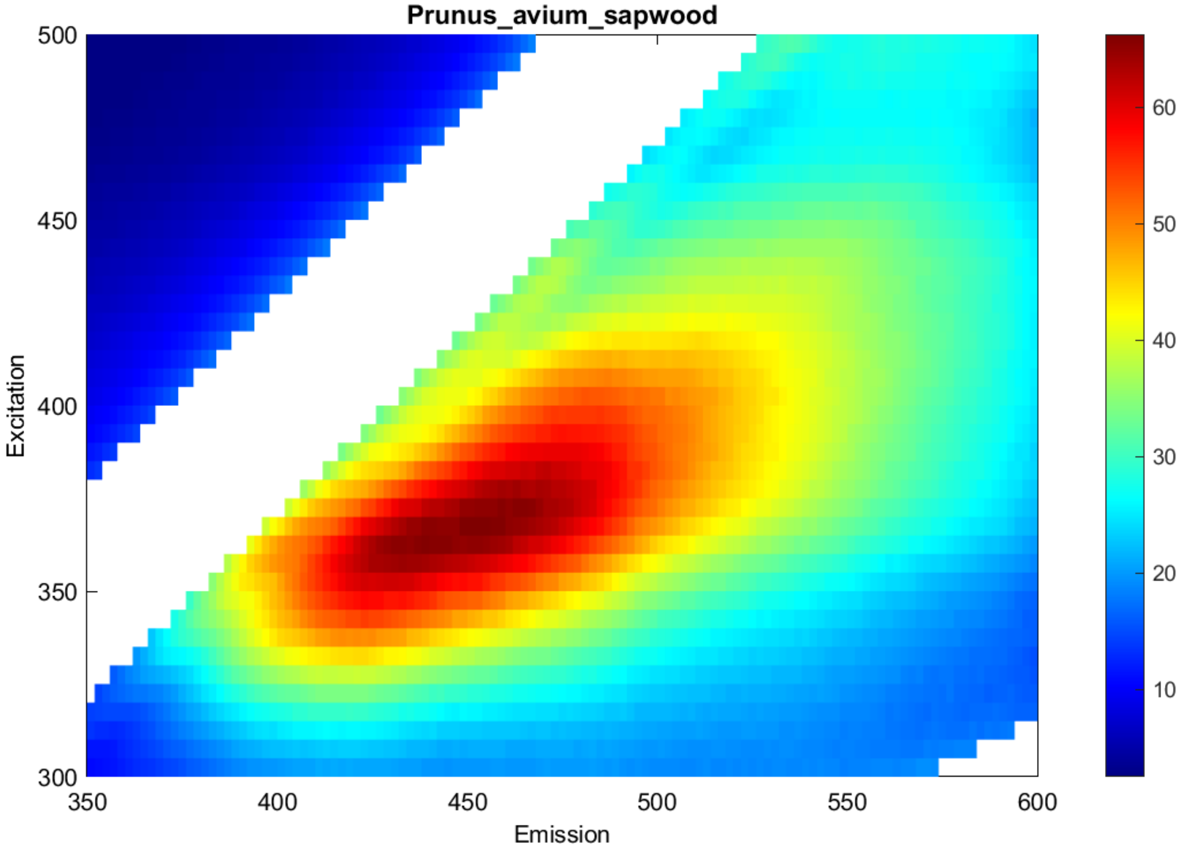

Figure S10: EEM of sample 10: untreated Prunus avium sapwood

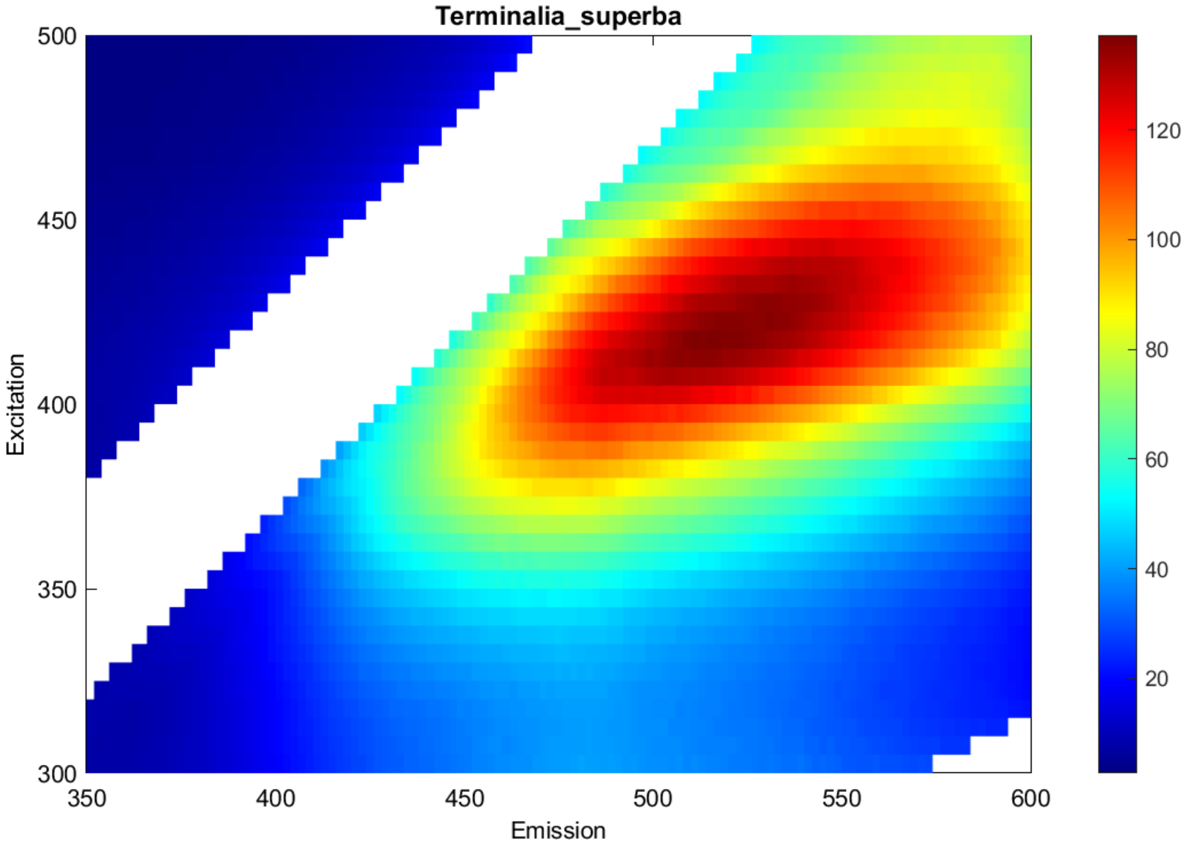

Figure S11: EEM of sample 11: untreated Terminalia superba wood

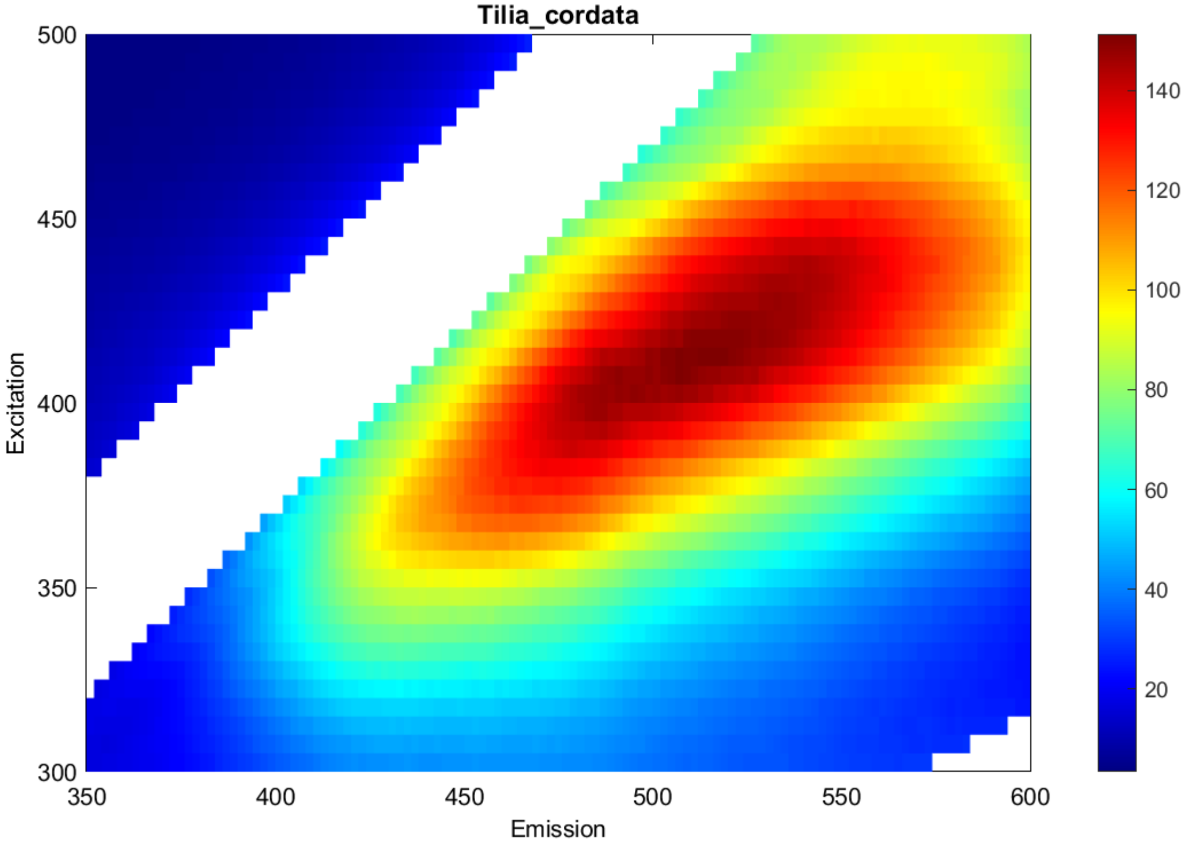

Figure S12: EEM of sample 12: untreated Tilia ssp. wood

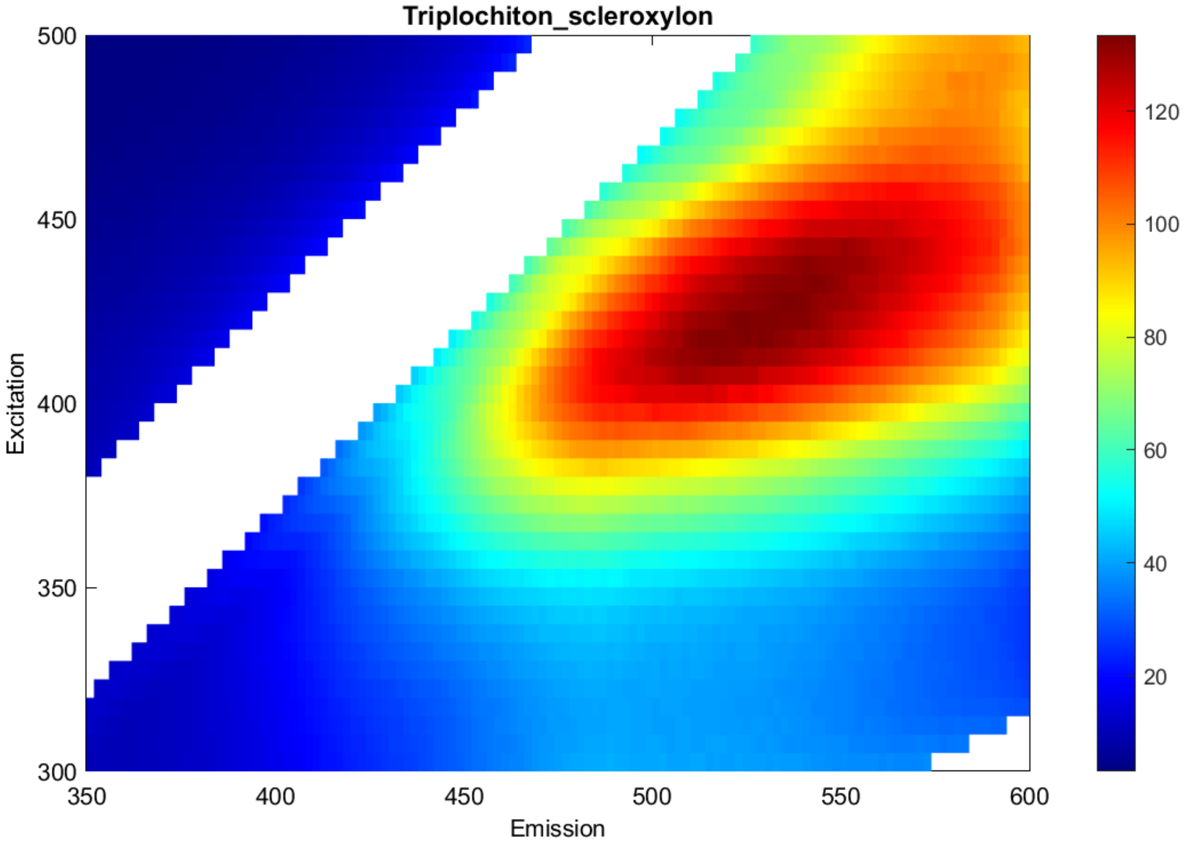

Figure S13: EEM of sample 13: untreated Triplochiton scleroxylon wood

Excitation-emission-matrices (EEMs) of treated wood:

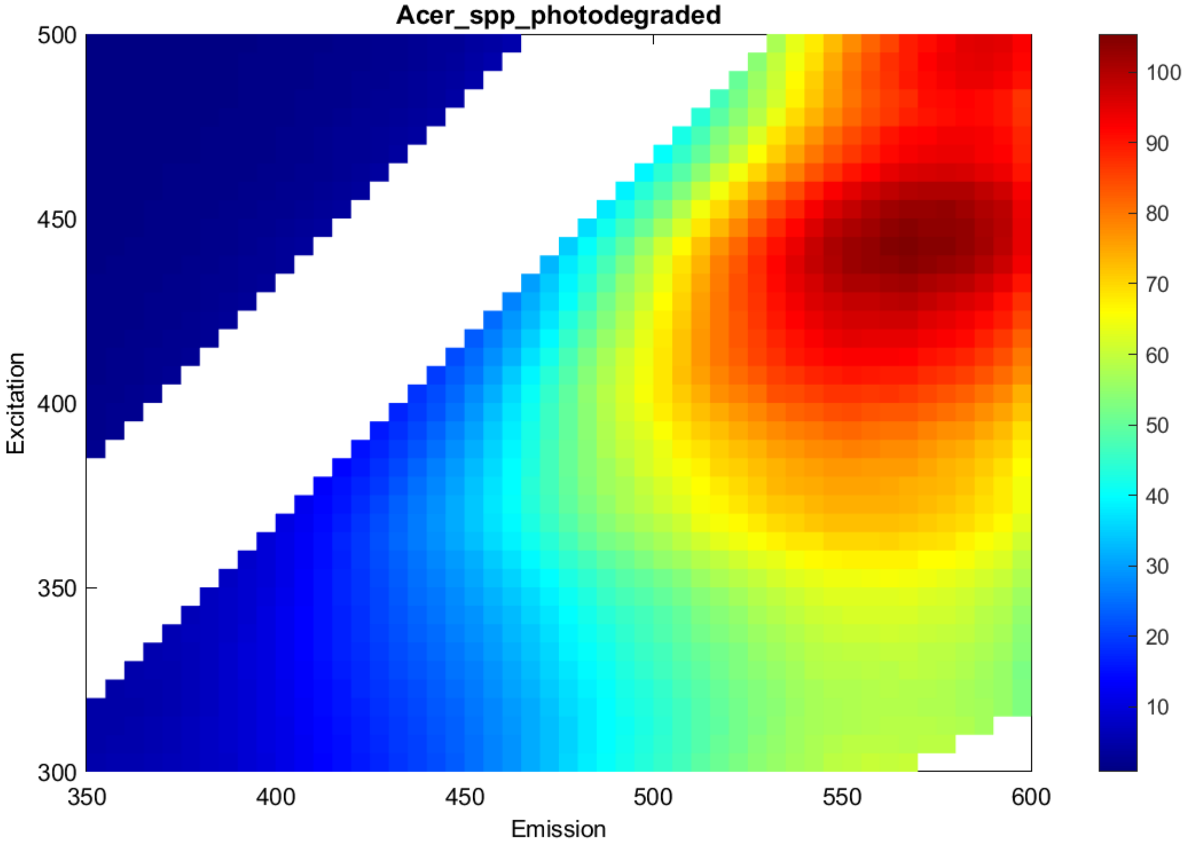

Figure S14: EEM of sample 14: Acer app. wood, photodegraded

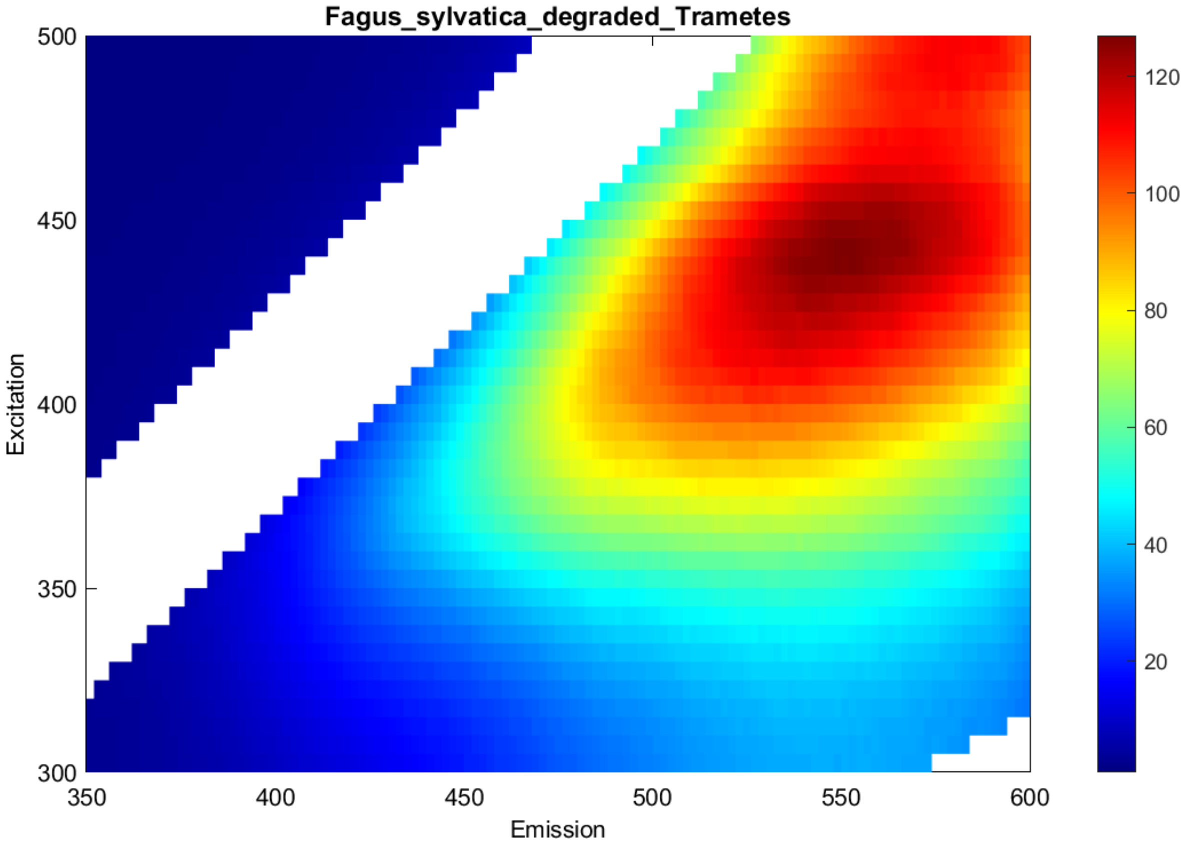

Figure S15: EEM of sample 15: Fagus sylvatica wood, degraded by Trametes versicolor

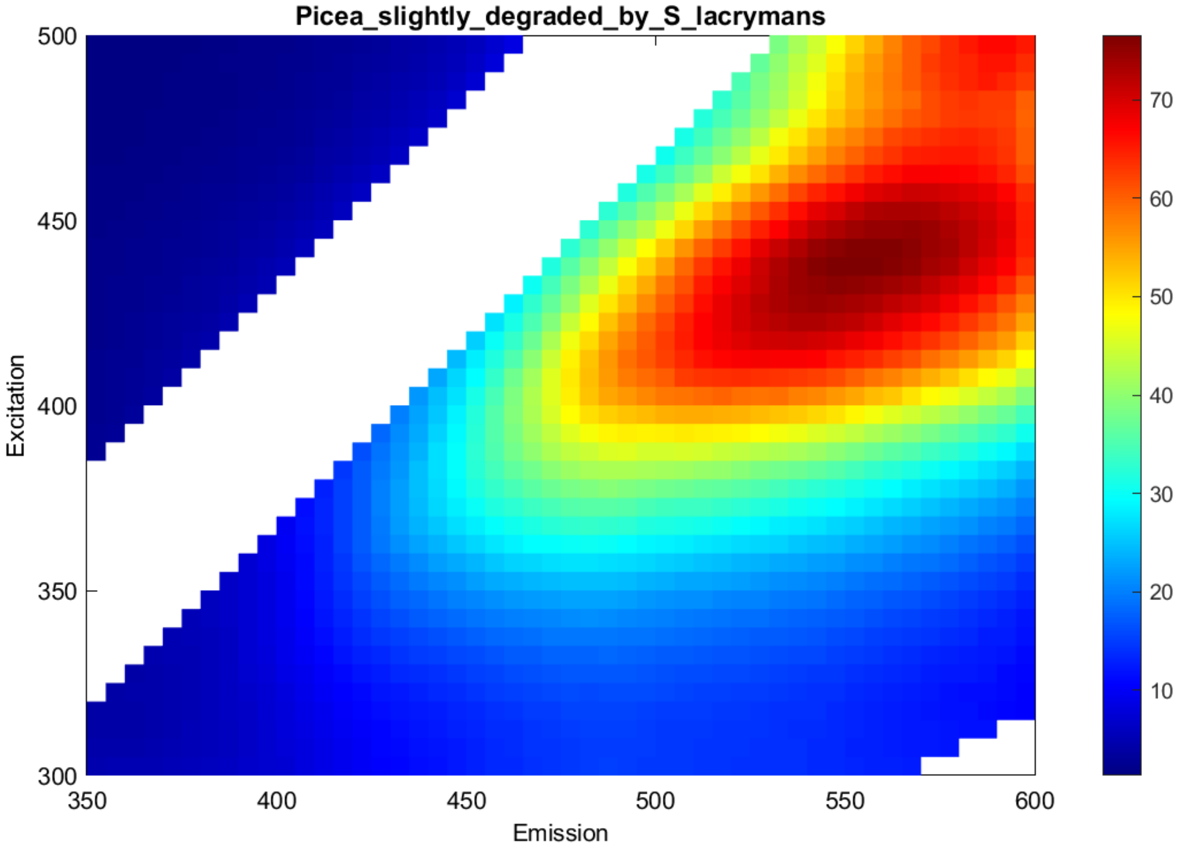

Figure S16: EEM of sample 16: Picea abies wood, slightly degraded by Serpula lacrymans

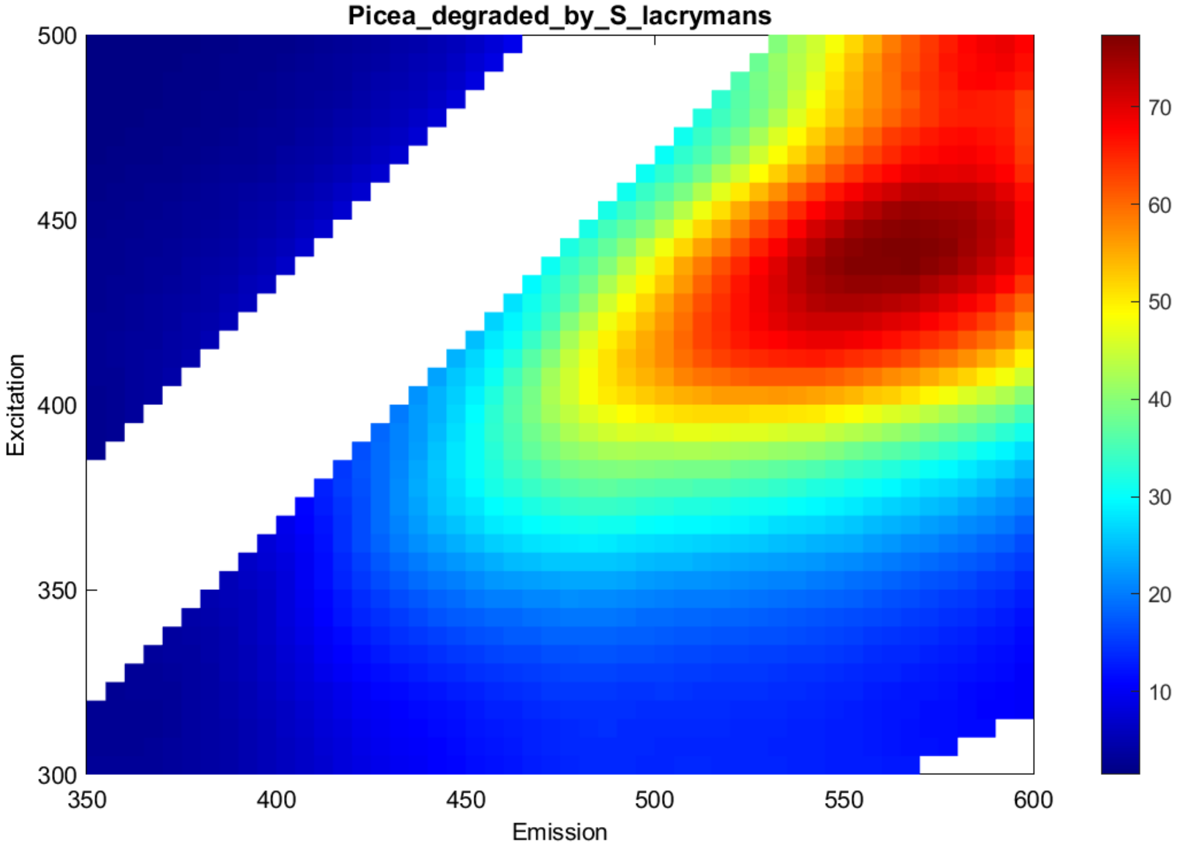

Figure S17: EEM of sample 17: Picea abies wood, degraded by Serpula lacrymans

Excitation-emission-matrices (EEMs) of polysaccharides and lignin:

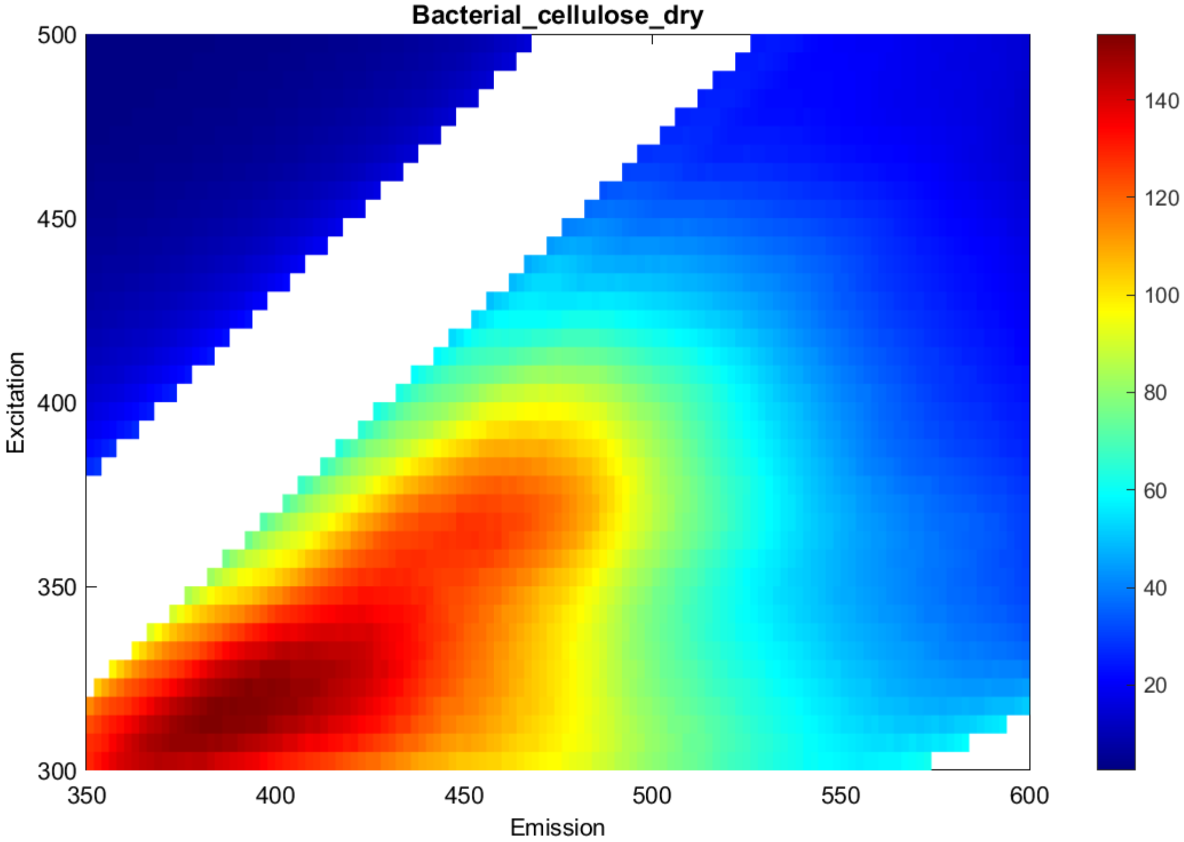

Figure S18: EEM of sample 18: bacterial cellulose, stored at room temperature at 40-50 % humidity

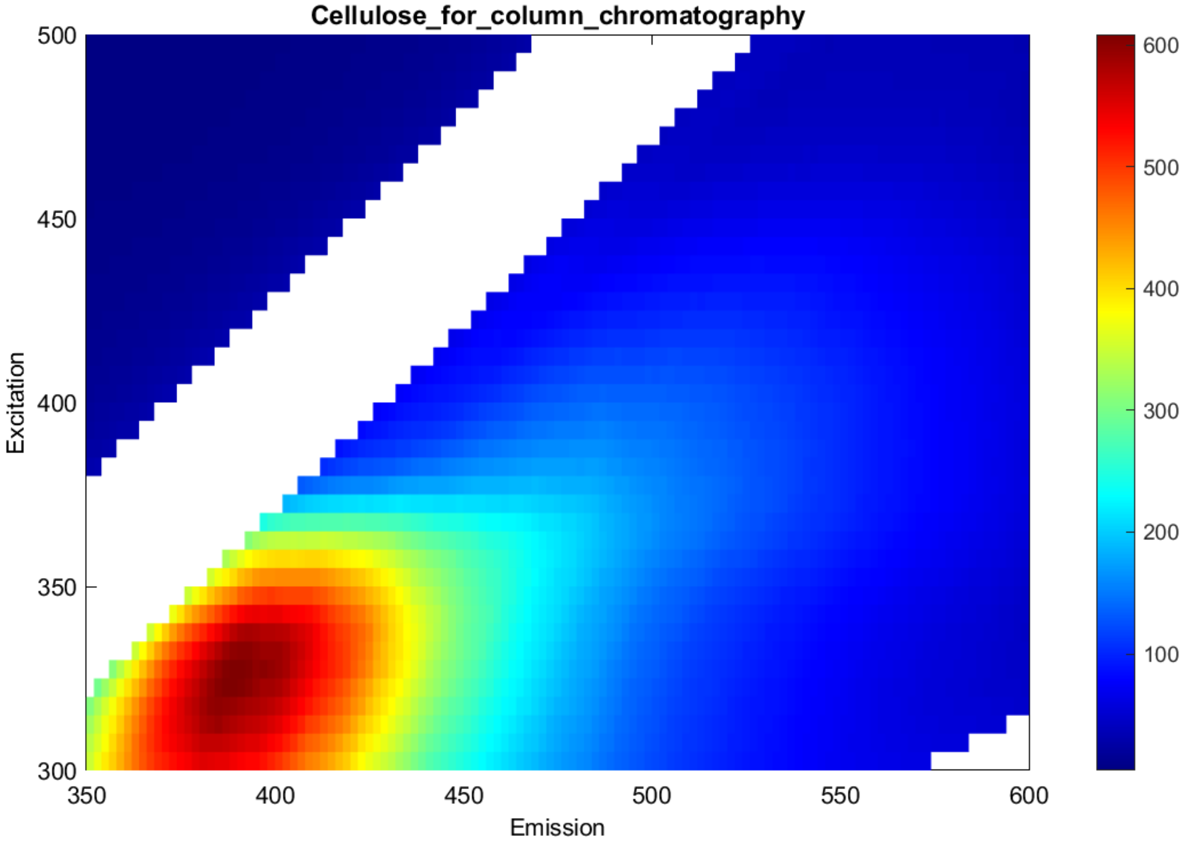

Figure S19: EEM of sample 19: cellulose for column chromatography

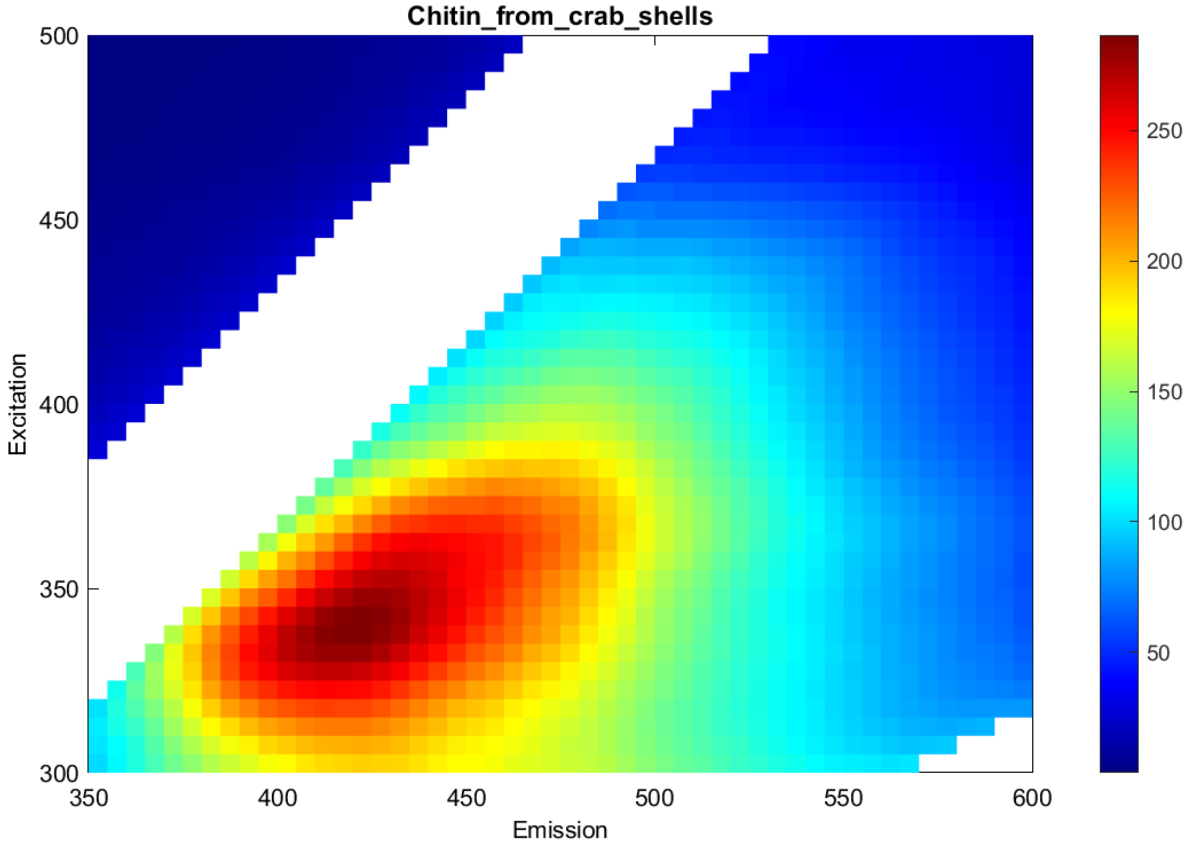

Figure S20: EEM of sample 20: Chitin from crab shells

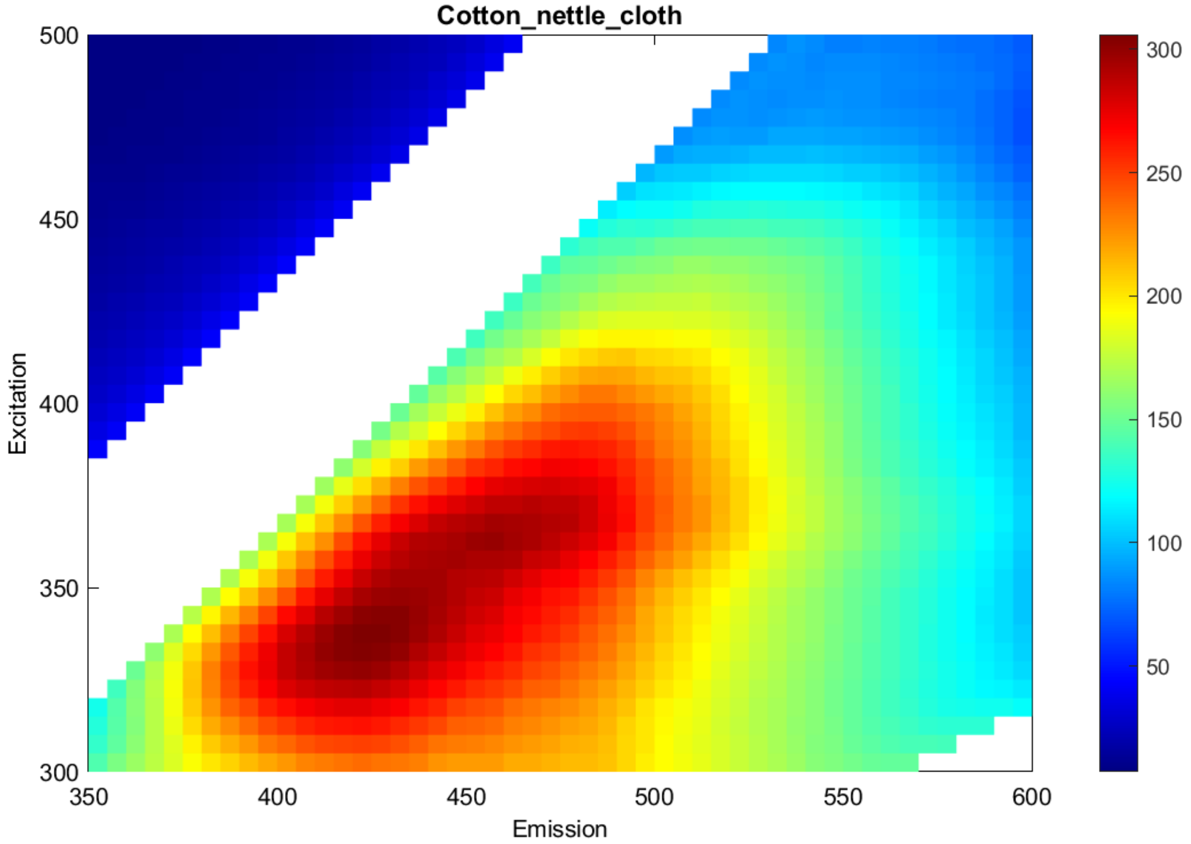

Figure S21: EEM of sample 21: commercial cotton nettle

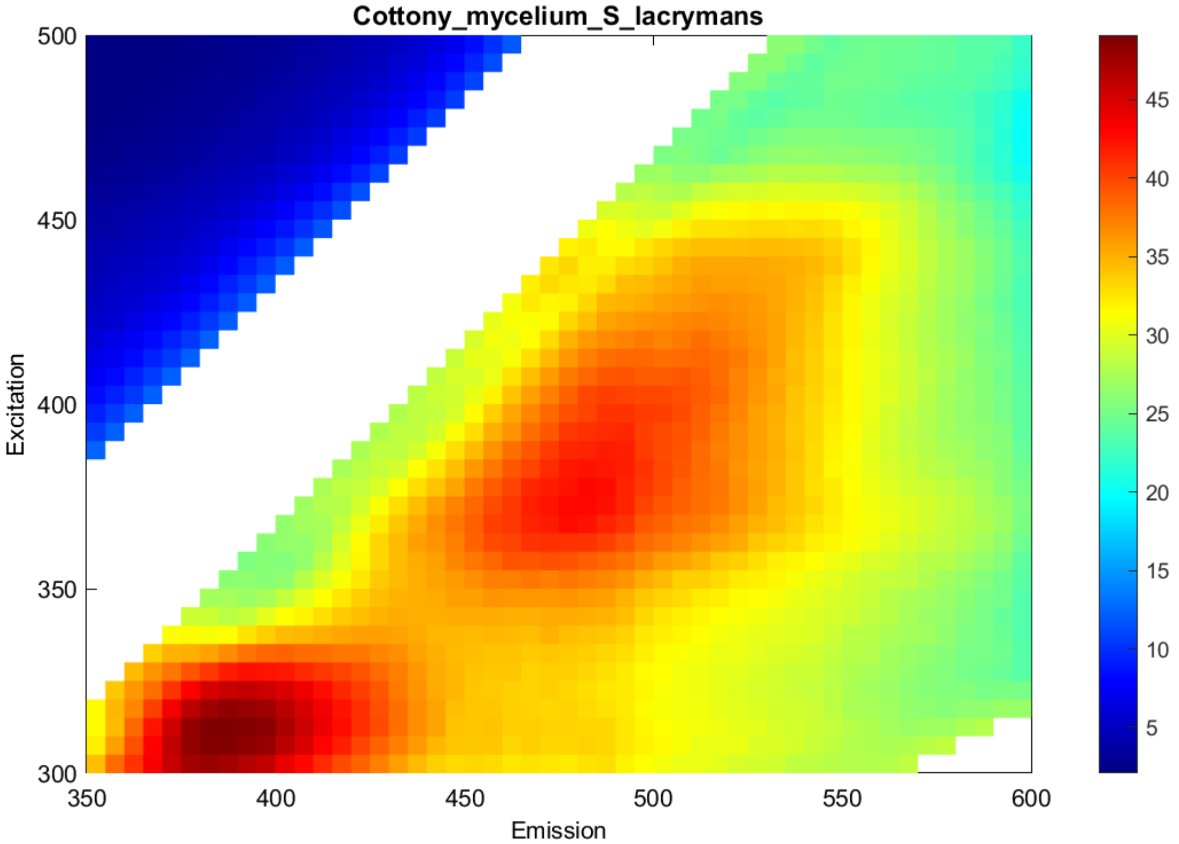

Figure S22: EEM of sample 22: Fluffy mycelium of Serpula lacrymans

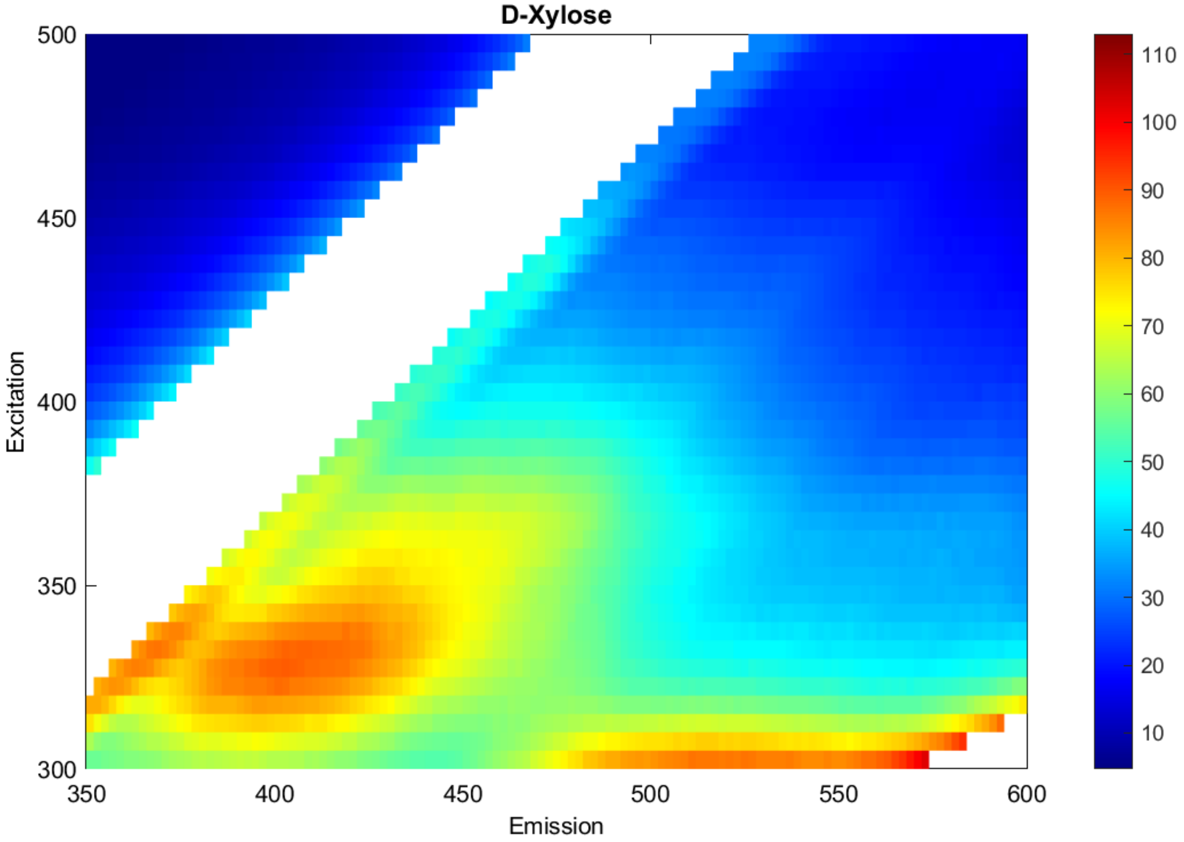

Figure S23: EEM of sample 23: D(+)Xylose

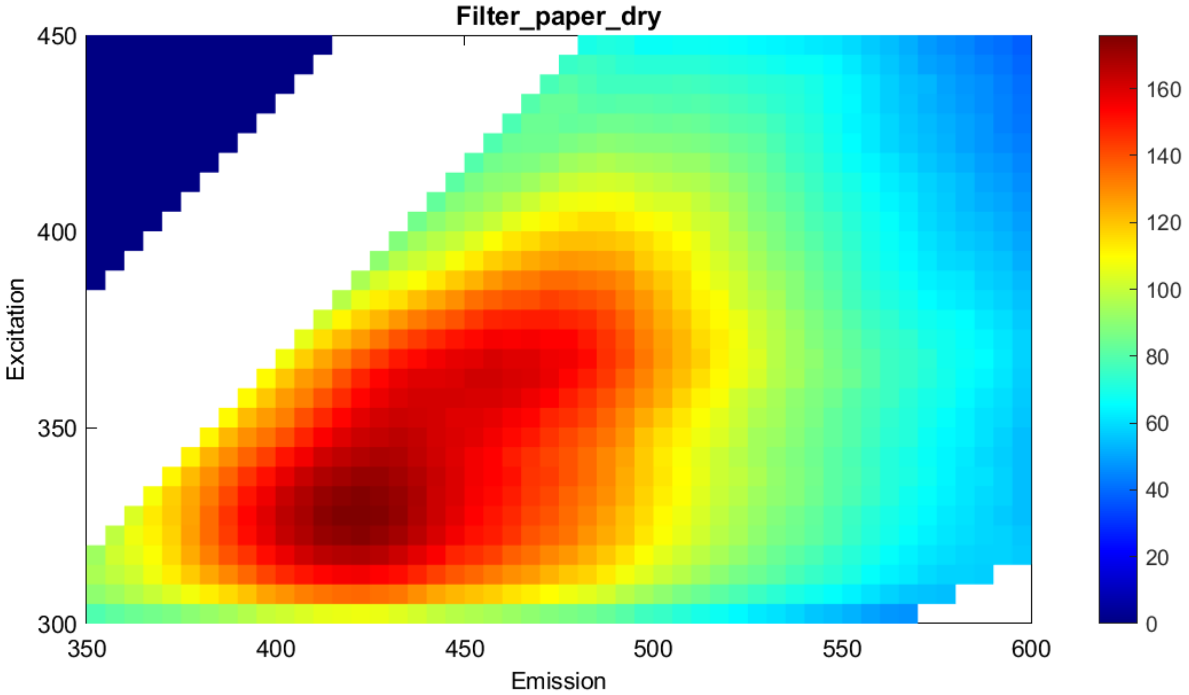

Figure S24: EEM of sample 24: Filter paper, stored at 2-3 % humidity

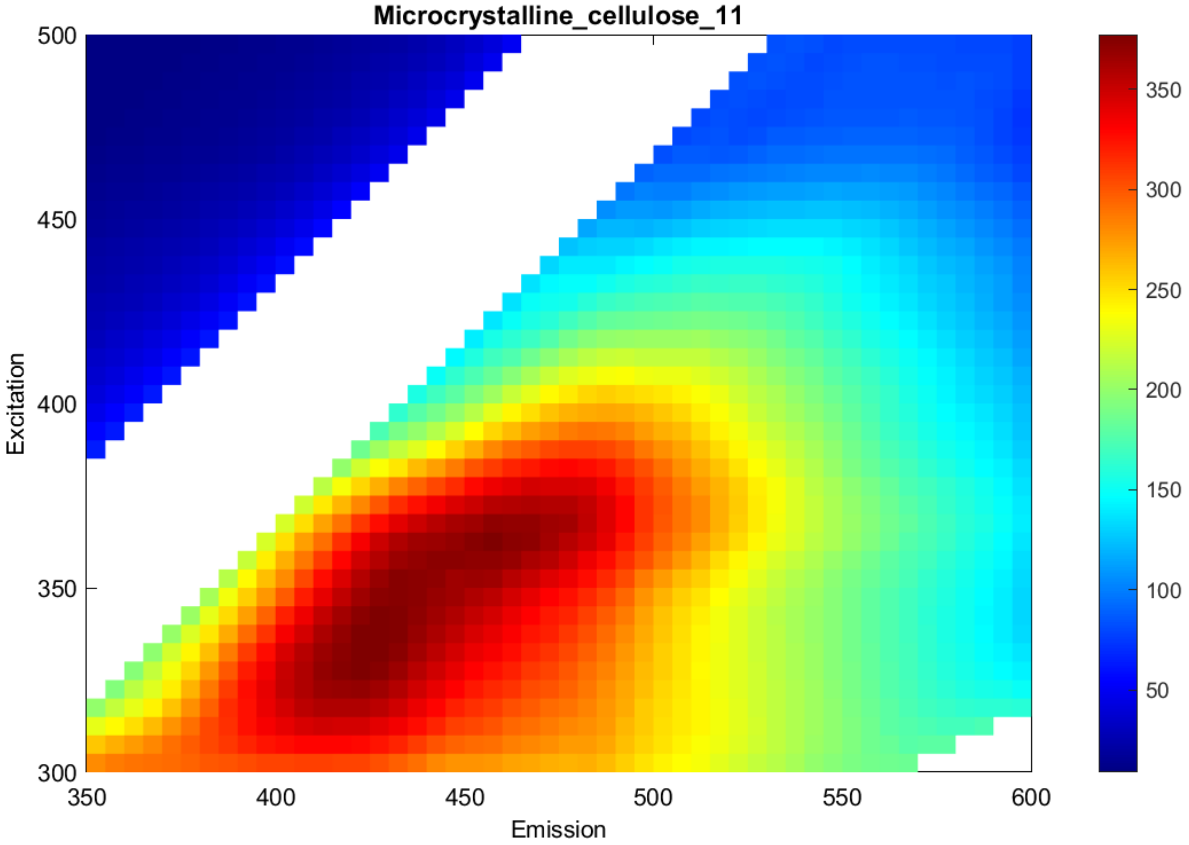

Figure S25: EEM of sample 25: microcrystalline cellulose, stored at 11 % humidity

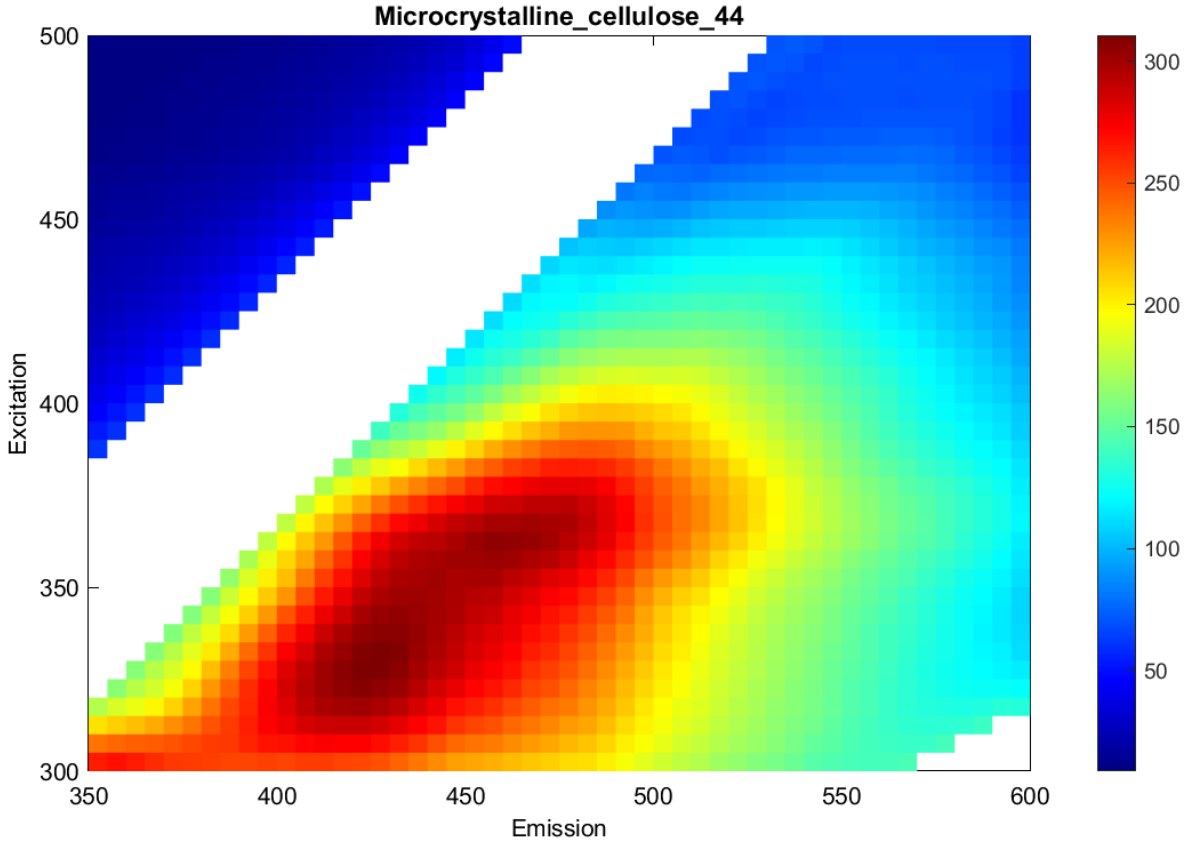

Figure S26: EEM of sample 26: microcrystalline cellulose, stored at 44 % humidity

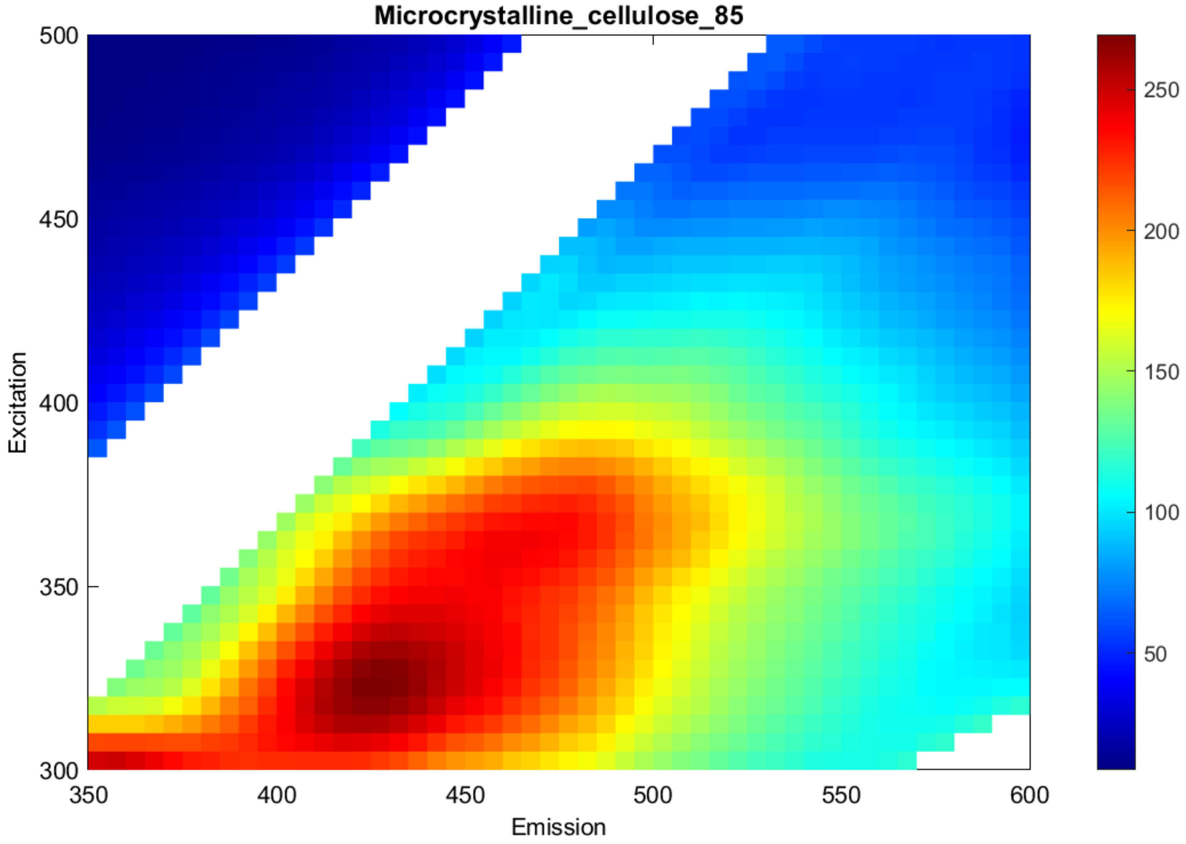

Figure S27: EEM of sample 27: microcrystalline cellulose, stored at 85 % humidity

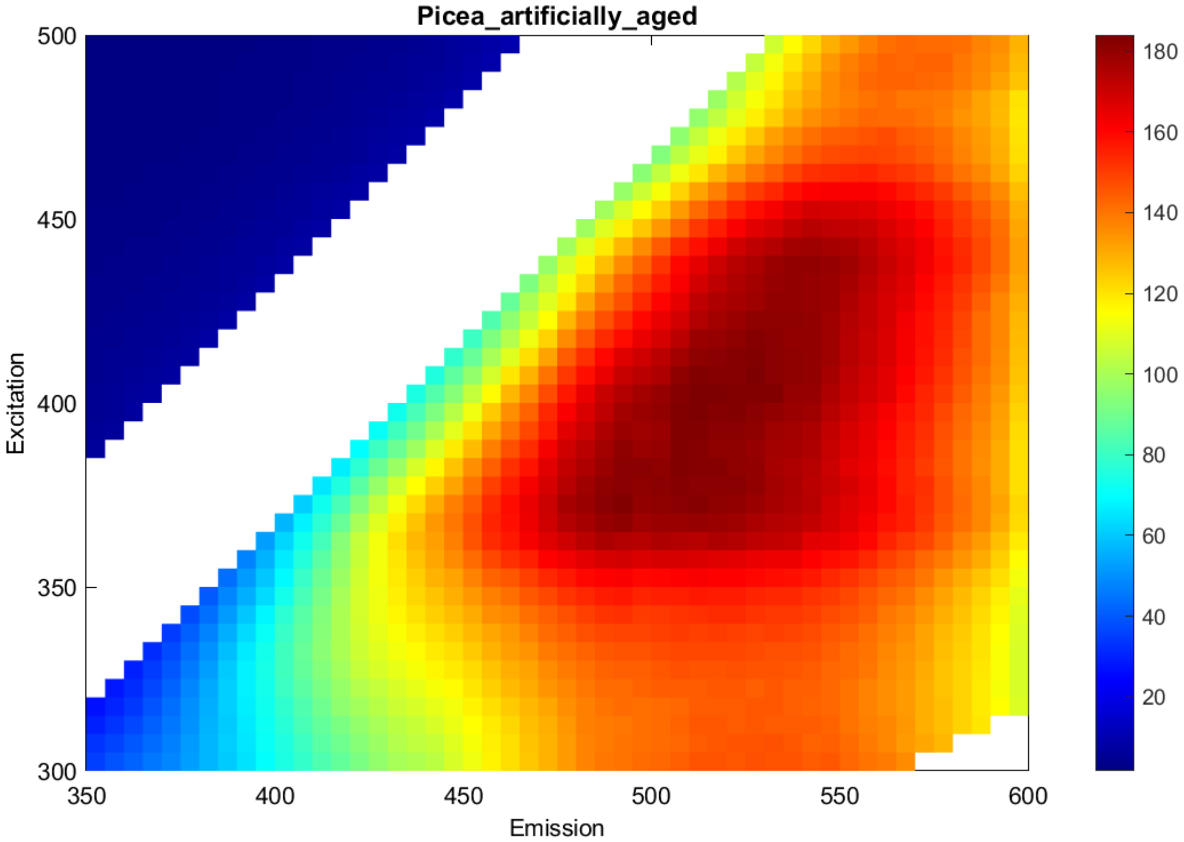

Figure S28: EEM of sample 28: artificially weathered Picea abies wood

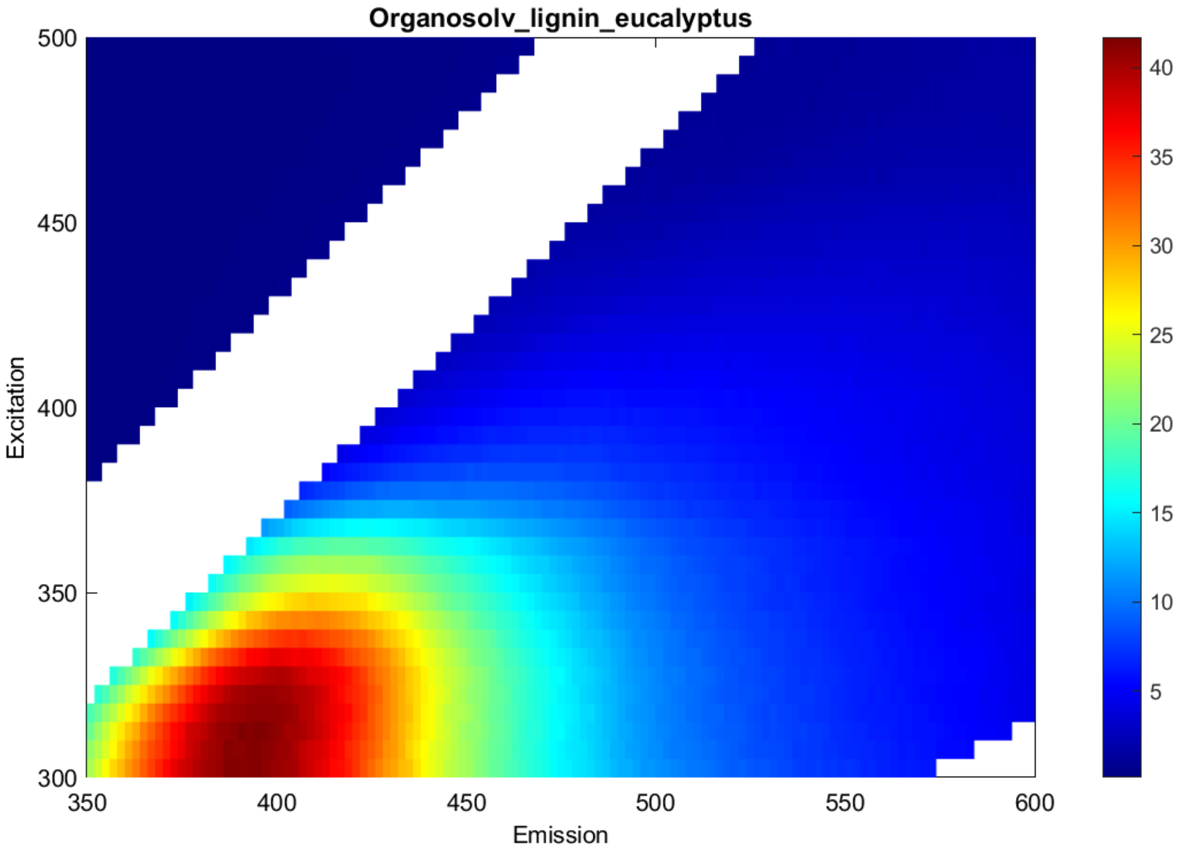

Figure S29: EEM of sample 29: 0.1 % solution of organosolv lignin in 60 % ethanol
